# Comparison of missing data handling methods for variant pathogenicity predictors

**DOI:** 10.1093/nargab/lqaf133

**Published:** 2025-10-14

**Authors:** Mikko Särkkä, Sami Myöhänen, Kaloyan Marinov, Inka Saarinen, Leo Lahti, Vittorio Fortino, Jussi Paananen

**Affiliations:** Blueprint Genetics Oy, 02150 Espoo, Finland; Department of Health Sciences, School of Medicine, Institute of Biomedicine, University of Eastern Finland, 70211 Kuopio, Finland; Blueprint Genetics Oy, 02150 Espoo, Finland; Blueprint Genetics Oy, 02150 Espoo, Finland; Blueprint Genetics Oy, 02150 Espoo, Finland; Department of Computing, Faculty of Technology, University of Turku, 20014 Turku, Finland; Department of Health Sciences, School of Medicine, Institute of Biomedicine, University of Eastern Finland, 70211 Kuopio, Finland; Blueprint Genetics Oy, 02150 Espoo, Finland; Department of Health Sciences, School of Medicine, Institute of Biomedicine, University of Eastern Finland, 70211 Kuopio, Finland

## Abstract

Modern clinical genetic tests utilize next-generation sequencing (NGS) approaches to comprehensively analyze genetic variants from patients. Out of millions of variants, clinically relevant variants that match the patient’s phenotype must be identified accurately and rapidly. As manual evaluation is not a feasible option for meeting the speed and volume requirements of clinical genetic testing, automated solutions are needed. Various machine learning (ML), artificial intelligence (AI), and *in silico* variant pathogenicity predictors have been developed to solve this challenge. These solutions rely on comprehensive data and struggle with the sparse genetic annotations. Therefore, careful treatment of missing data is necessary, and the selected methods may have a huge impact on the accuracy, reliability, speed and associated computational costs. We present an open-source framework called AMISS that can be used to evaluate performance of different methods for handling missing genetic variant data in the context of variant pathogenicity prediction. Using AMISS, we evaluated 14 methods for handling missing values. The performance of these methods varied substantially in terms of precision, computational costs, and other attributes. Overall, simpler imputation methods and specifically mean imputation performed best.

## Introduction

### Genetic variant pathogenicity prediction

Next-generation sequencing (NGS) technologies have greatly improved the scalability of genetic sequencing in both research and clinical settings. Whole-exome sequencing (WES) and whole-genome sequencing (WGS) are now becoming standard methodology, and detection of variants is now feasible in a much broader set of loci than previously. However, the large numbers of variants from each sample present problems especially in clinical contexts. The often time-critical process of identifying clinically relevant genetic variants requires the manual and painstaking exploration of long variant lists. Highly accurate computational tools could further improve the filtering or prioritization process.

Many tools applicable for this purpose already exist. Though often developed for use in research contexts, many are also used in clinical variant interpretation (see ACMG/AMP guidelines) [1]. *In silico* variant effect and pathogenicity prediction tools have been developed to inform the selection of variants for further testing (e.g. SIFT [2], PROVEAN [3], MutationTaster2 [4, 5], LRT [6] and FATHMM [7]). Similarly, gene and variant prioritization tools have been developed to rank variants for exploration (e.g. VAAST [8], PHEVOR [9], FunSeq [10], PHIVE [11] and Phen-Gen [12]). Peterson *et al.* [13], Niroula & Vihinen [14], and Eilbeck *et al.* [15] discuss the already diverse set of prediction tools.

The primary feature of most deleteriousness prediction tools is a metric of sequence conservation supplemented with additional features, sometimes derived from outputs of previously developed tools [13]. Machine learning (ML) methods including such input features form the class of *metapredictors* or *ensemble predictors*.

Metapredictors often report high performance [16, 17] and are applicable to a wider range of variants by simultaneously utilizing inputs describing variants with different predicted consequences, e.g. by combining missense as well as splicing effect prediction tools. Examples of metapredictors are REVEL [18], CADD[19, 20], DANN [21], Eigen [22], PON-P [23], and MetaSVM and MetaLR [24].

Even tools whose use cases match can differ in their domain of prediction due to different data and methods. Building a metapredictor that incorporates several existing tools without restricting domain of prediction thus requires handling input features with *missing values*. Missing values can also arise in features representing experimentally obtained data. For example, allele frequency information will have missing values for variants that were not observed in the aggregated cohorts.

#### Missingness handling in existing metapredictors

Strategies for missingness handling vary widely between different existing metapredictors. REVEL [18] uses k-NN imputation when a variant’s missingness is ≤50%, and mean imputation when missingness is >50%. CADD [19, 20] and DANN [21] use a mix of manually defined default values to replace missing values, with added missingness indicators for certain features, and mean imputation for genome-wide measures (see Supplementary information for [19]). M-CAP replaces missing values with constants representing the maximally pathogenic prediction for each component tool [25]. Eigen [22] utilizes separate strategies for training and test phases, and builds several weighted linear combinations of its features depending on variant type, requiring only annotations applicable to a specific variant to be available. Learning the weights is based on pairwise correlation, which can be estimated in the presence of some missing values. In the test phase, Eigen performs mean imputation for features that are applicable to the specific variant. KGGSeq ignores any variants that has missing values in its features [26]. PRVCS [27] removes variants with missing values in the training phase and replaces missing values of a feature by its population mean in the test phase.

### Missingness handling in prediction

The traditional classification of missing data processes to *missing completely at random* (MCAR), *missing at random* (MAR), and *missing not at random* (MNAR), depending on whether the missingness probability depends on the observed or unobserved values of the dataset. The validity of imputation techniques in statistical inference depend on which of these classes are assumed to appear in the data [28]. Further, using even highly accurate single imputation methods will cause underestimation of standard errors [29, subchapter 2.6]. The uncertainty can be incorporated into the estimates by *likelihood-based approaches* or by *multiple imputation* (see [28]).

However, Sarle [30] notes that ‘The usual characterizations of missing values as MAR or MCAR are important for estimation but not prediction’, and Ding & Simonoff [31] provide evidence in support of this statement in the use of classification trees. In an interesting reversal, the presence of *informative missingness* [30, 31] in the data (i.e. missingness being dependent on the response variable conditional on observed values) may lead to improved predictive accuracy compared to complete data [31]. The approach and concerns of a researcher building a model for the prediction of a response variable differ significantly in both approach and concerns to that of the statistician looking to assess whether data supports a hypothesis (see [32, 33]). However, many missingness handling methods are developed for use in statistical inference, and their applicability to prediction is still unclear.

### Causes for missingness in variant annotation data

Most missingness in variant annotation data can be attributed to the following causes:

- Insufficient information related to the variant
- Inapplicability to the variant

For an example of the first, consider protein function change prediction tools based on multiple alignment. When sufficient matches to the input sequence are not found, the tool has no information on which to base its estimates. Another example is values estimated from scientific studies (e.g. allele frequencies, functional properties of proteins or expression levels of transcripts). Variants that are unobserved in the cohorts, excluded from genotyping microarrays, or inside hard-to-sequence loci will have no observed allele frequency.

The second cause refers to attempts to annotate a variant with information that is inapplicable to its predicted molecular consequence. For example, trying to predict or measure change in protein function for an intronic variant.

Uttermost caution must be exercised in performing any hypothesis testing on imputed data with the latter missingness cause. However, predictive models can still make use of such data to learn patterns in other features. For example, patterns in conservation scores may be better estimated from a full dataset including variants of many types, even if the imputation of a protein function prediction produces a meaningless value for an intronic variant. The improvement in performance from the patterns might in some cases exceed the decrease due to noise introduced by the imputation. The decrease in performance can further be mitigated if the model type is chosen to be flexible enough to learn to disregard features with meaningless imputation depending on variant type. One way to facilitate this is to use missingness indicator augmentation [34], where additional missingness indicator features are added, after which each feature is imputed with zero. The downside of missingness indicator augmentation is doubling of the dimensionality of the feature space in the worst case. We include missingness indicator augmentation in our comparison.

A domain expert could also in some cases choose an *a priori* appropriate value to reflect a biological interpretation, e.g. assign zero to missing values in the example of measured protein change for variants outside coding regions. We utilize this approach for certain features. However, this is cumbersome for large numbers of features, and essentially corresponds to constant imputation on the feature.

Missingness due to mismatch to molecular consequence could be avoided by training separate models on each consequence and using only features fully applicable to each consequence. A general approach in this vein is to use reduced models [35] or reduced-feature models [36], in which one trains a separate predictor for each combination of missing features using only the available features for each subset. This is observed by Saar-Tsechansky & Provost to perform well consistently [36]. However, a naïve implementation of reduced models easily leads to extremely high computational time and storage requirements, and the hybrid and on-demand approaches described by Saar-Tsechansky and Provost [36] are not trivial to implement. Note that partial application of this paradigm via training multiple models by training different models depeding on molecular consequence would still contain missing values due to the first cause, and thus still require additional missingness handling. This solution also requires each consequence category to have sufficiently many observations to train a classifier.

### Aim

The high prevalence of missing values in annotated variant data implies that missingness handling will have a major role in the performance of variant pathogenicity metapredictors and ML/AI based variant classification and interpretation tools. Our aim is to identify missingness handling methods most likely to enable good performance in this context. For this purpose, we compare missingness handling methods by difficulty of implementation, by required computation time, and by classification performance on treated data.

We focus on the comparison of imputation methods as well as the missingness indicator augmentation method, as they are the most generally applicable and allow use of any ML/AI method on the treated dataset.

We limit our evaluation to imputation of numerical features, as categorical features have a relatively natural treatment through an additional category denoting missingness. In addition, we choose to focus on building a single classifier for single-nucleotide variants (SNVs) and small indels, rather than separate classifiers per variant consequence class.

To further improve the relevance of our results, we utilize ClinGen [37], an expert-reviewed subset of ClinVar [38–40] as the basis of our analyses.

To support the experiments and enable possible future work, we also implement and present a framework called AMISS implemented in the R language [41] to automatize common tasks between the experiments.

## Materials and methods

We present a framework implemented in R that enables comparison of imputation methods by their contribution to the performance of machine-learning classification of variant pathogenicity. The framework preprocesses variant data to a usable format, performs imputation, trains a classifier, and computes relevant performance statistics.

This framework is used to perform three experiments:

- an additional missingness experiment, where additional missing values are generated in the dataset several times, and the framework is used on each resulting dataset, described in the ‘Additional missingness experiment’ section,
- a cross-validation experiment, where the framework is used on datasets produced using repeated random sub-sampling, described in the ‘Cross-validation experiment’ section, and
- a main experiment, where the framework is used once with more comprehensive hyperparameter ranges for imputation methods, described in the ‘Main experiment’ section.

### Framework

The AMISS framework performs imputation and classifier training and evaluation on a preprocessed training and test dataset pair. The preprocessed training set is used to filter out features that have insufficiently unique values or that are heavily correlated with some other feature, and this filtering is matched on the test set (see the ‘Features’ section).

Each imputation method is used on the training set, producing at least one imputed dataset for each combination of hyperparameters (see below), and classifiers are trained on each dataset.

To avoid obfuscation of the effect of imputation in consequence classes where class imbalance is too large, we removed variants with consequences where either class had <5% of overall variants of that consequence. See the description for Supplementary Table S2 for details.

To minimize issues due to singular matrices with some imputation methods [e.g. multiple imputation by chained equation (MICE) linear regression, MICE predictive mean matching], we removed features with fewer than 1% unique values. For feature pairs with high correlation (Pearson correlation coefficient >0.9), we kept only one of the features. Removal of features with few unique values or high correlation is performed as part of the training process, just before imputation, since they may be affected by introduction of additional missing values in the additional missingness experiment (see the ‘Additional missingness experiment’ section). However, we chose to use the same restricted feature set for all imputation methods for simplicity and comparability.

To maximize the performance of each imputation method, hyperparameter grids were defined for each method. For the additional missingness and cross-validation experiments we decided to save computational time by using fewer hyperparameter configurations, sampling a maximum of eight hyperparameter configurations for each imputation method used on a dataset. The hyperparameter grids are described in Table 5.

**Table 1.** Features imputed with default values

Feature name	Feature interpretation	Default value
motifDist	‘Reference minus alternate allele difference in nucleotide frequency within an predicted overlapping motif’ [19, Supplementary information]	0
gnomAD_exomes_AF	gnomAD allele frequency from exomes	0
gnomAD_genomes_AF	gnomAD allele frequency from genomes	0

**Table 2.** Dataset composition

	Total	Training set	Test set
Total	3736	2606	1130
Positive	1564	1088	476
Negative	2172	1518	654

**Table 3.** Mockup of data after preprocessing

Variant	SIFT	DNase-seq	gnomAD AF	mirSVR	Non-synon.	Intronic	…
1:215625828:T:C	NA	0.3126	0.0	NA	0	0	
2:39022774:T:C	0.001	0.2607	4.070e−06	NA	1	0	
2:47410217:G:A	0.033	0.3818	0.0	NA	1	0	
2:47797737:C:T	NA	0.5263	0.0	NA	0	1	
13:32399139:A:G	NA	0.0750	0.0	−0.1513	0	0	

Variant identification information is not used in imputation or training. gnomAD allele frequency has a value exactly 0 on rows 1, 3, 4, and 5 due to *a priori* imputation.

**Table 4.** Included imputation methods

Imputation method	Description	Implementation
Zero imputation	Replace by 0	Custom
Maximum imputation	Replace by maximum observed value within feature	Custom
Minimum imputation	Replace by minimum observed value within feature	Custom
Median imputation	Replace by median observed value within feature	Custom
Mean imputation	Replace by mean observed value within feature	Custom
Outlier imputation	Given observed values *F*_obs_ of feature *F*, replace by |max(*F*_obs_) − min(*F*_obs_)| × 10	Custom
Missingness indicator augmentation	For each feature, perform zero imputation and create a binary feature indicating original missing values	Custom
Predictive mean matching	Sampling from observed values similar to a predicted value	pmm in package mice
Random forest (RF)	Predict values using RF	rf in package mice
Linear regression	Predict values from a linear regression model	norm.predict in package mice
Bayesian linear regression	Predict values from a linear regression model with added uncertainty modeling noise and variability of parameter estimates	norm in package mice
Bayesian principal components analysis (BPCA) [55]	Predict values via BPCA	bpca in package pcamethods[57]
k-NN	Predict values as mean of *k* nearest neighbors wrt other features	knnimputation in package dmwr [58]
MissForest [56]	Predict values using RF	missforest in package missforest [59]

**Table 5.** Statistics on hyperparameter grids for imputation methods

Method	Varied	Number of
	hyperparameters	combinations
MICE PMM	donors, ridge, matchtype	180
MICE regression	None	1
MICE Bayes regression	None	1
MICE RF	ntree	36
k-NN	k	5 (effectively 2)
BPCA	npcs, maxsteps	112
MissForest:	mtry, ntree	12
Simple methods	None	1 each

For k-NN, only two values of k succeed, see the ‘Included missingness handling methods’ section.

Categorical features were transformed to dummy variables with an extra category denoting a missing value. It is important to note that thus no imputation methods designed specifically for categorical features were tested, and categorical features were not treated by the used numerical imputation methods.

For each method, after training set imputations and classifier training, we select the classifier (or classifier set, for multiple imputation) with the highest performance with respect to (mean) MCC (Matthews' correlation coefficient) on the imputed training set(s). The associated imputation hyperparameters and the classifier itself are stored. The stored classifier’s performance is then estimated on the test set, which is imputed using the stored hyperparameters.

**Figure 1.**
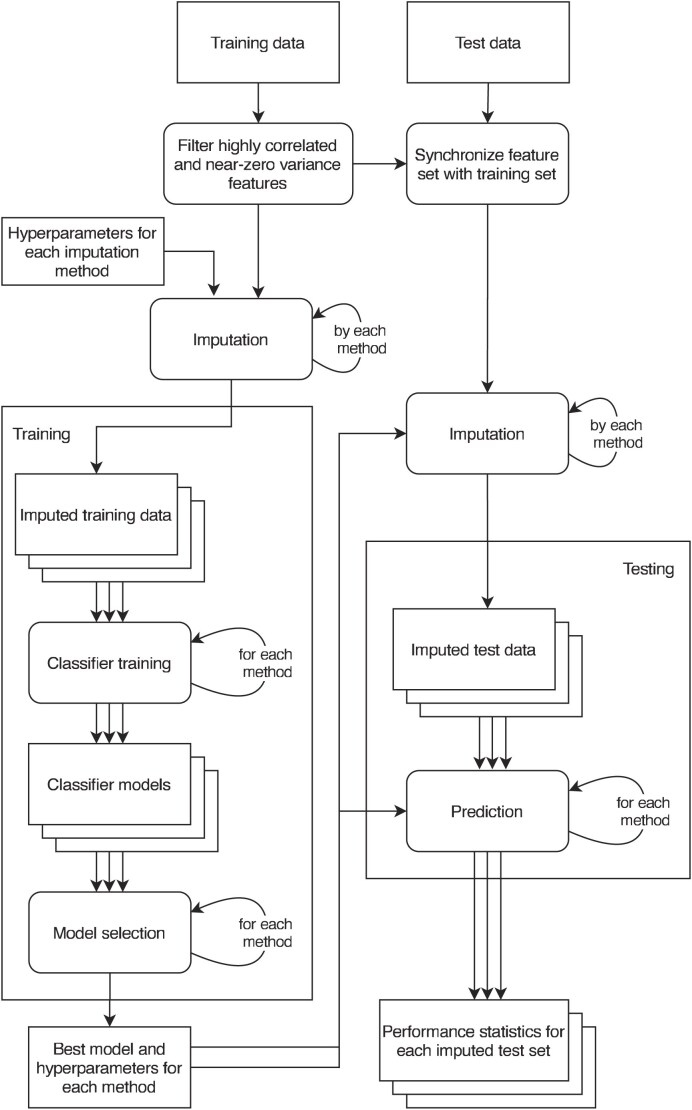
Flowchart depicts the flow of data in the framework. In the diagram square boxes represent datasets, and stacked square boxes represent a set of multiple datasets. Rounded boxes represent actions, and arrows depict the flow of data. Arrows from an action to itself represents repeating of the action. Multiple arrows from multiple datasets represents the flow of a set of datasets. Features are filtered on the training data according to their correlations and variance, and the feature set of the test data is synchronized to match the filtered feature set. Afterwards, the training data is imputed using each imputation method, with each hyperparameter configuration. In the main experiment, multiple imputation methods and MissForest are set to produce 10 imputed datasets per hyperparameter configuration. Only one dataset is set to be produced in the additional missingness and cross-validation experiments. Every dataset is then used to train a classifier, and for each imputation method the best-performing hyperparameter configuration is selected by the training-set performance of the corresponding classifier. For multiple imputation methods, the mean performance with respect to MCC of the corresponding classifier set is used. The test set is then imputed with the selected hyperparameter configuration for each method, and corresponding classifier is used to predict on the imputed test set(s). Classifier performance is then evaluated on each set of predictions.

The process is depicted visually in Fig. 1.

We restricted our analysis to two common classification methods: logistic regression (LR), a standard statistical method for two-class problems, and random forest (RF) [42], a highly flexible machine-learning method. Both methods have been used in existing variant pathogenicity predictors (e.g. KGGSeq [26] and later versions of CADD [20] utilize LR, while e.g. MutPred [43], PON-P [23], REVEL [18], and Meta-SNP [44] utilize RF).

LR is used with the base R glm, and RF is used via the package randomForest [45]. Both are trained and applied to test data via functionality from the caret package [46].

The RF was trained using the out-of-bag performance for model selection, optimizing over the mtry parameter across values 7, 15, 23, 31 and 39. glm does not offer any tuning parameters.

### Data

The dataset consists of ClinGen[37] expert-reviewed SNVs from ClinVar [38–40], downloaded on 28 June 2019. We assume that all variants have been classified where they arose as individual variants, and any multinucleotide variants would have been submitted as one variant, and thus that the variants are independent. We annotated the variants using the Ensembl Variant Effect Predictor (VEP) [47] version 96 with Ensembl transcripts and dbNSFP3.5 [48, 49]. We selected and retained transcripts annotated canonical by VEP for each variant and removed the other transcripts and their associated values, when present. Variants whose canonical VEP-annotated transcript ID did not match that from dbNSFP were discarded. In addition, we incorporated annotations used by CADD [20], matching them by transcript to the VEP-annotated Ensembl transcripts. At this stage, the dataset contained 12282 rows.

### Preprocessing

**Figure 2.**
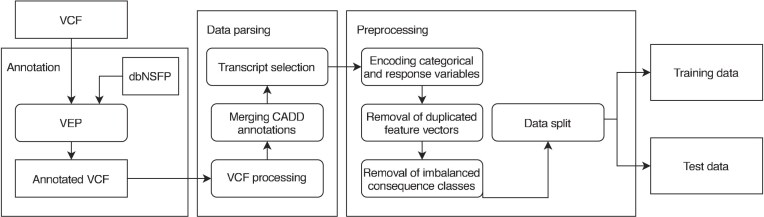
Preprocessing is divided into annotation, data parsing, and preprocessing steps. The VCF (Variant Call Format) file is first annotated using VEP to incorporate values from dbNSFP3.5. In data parsing, the annotated VCF is read and parsed, CADD annotations are merged, and canonical transcripts are selected. In preprocessing, categorical variables and response variables are encoded, duplicated feature vectors are removed, variants in highly imbalanced consequence classes are removed, and the dataset is split into training and test sets. Some preprocessing actions are deferred to the training phase, as their results may change due to additional generated missing values.

The overall preprocessing process is depicted in Fig. 2.

#### Features

The initial feature set was defined manually to include a variety of traditional tools and annotations from both dbNSFP3.5 and the annotation set for CADD while excluding any metapredictors from the feature set, and includes 47 numerical and 3 categorical features (listed in Supplementary Table S3). For histograms of observed values, correlations between numeric features and correlations to the positive outcome indicator for each feature, see Supplementary Figs S9 and S11–S19. Some variants may have equal (i.e. duplicated) feature vectors. We removed all except one variant from each equivalence class formed by duplicated feature vectors (*N* = 320 removed). We also chose to drop variants of uncertain significance (*N* = 1157) from the dataset.

For features where the missingness implied the default value *a priori*, missing values were replaced by default values. These features are listed in Table 1.

#### Data split

The data was randomly split into training and test subsets, with 70% (*N* = 7536) of variants in the training set and 30% (*N* = 3269) of variants in the test set.

We formed a binary outcome vector by defining variants classified as pathogenic or likely pathogenic to belong to the positive class (*N* = 5090 in the training set, *N* = 2218 in the test set), and variants classified as benign or likely benign to belong to the negative class (*N* = 2446 in the training set, *N* = 1051 in the test set).

We removed variants with consequences where either class had <5% of overall variants of that consequence. This lead to removal of 4930 variants from the training and 2139 variants from the test set.

The final dataset contains 3736 variants divided into a training set with a total of 2606 variants, of which 1088 belong to the positive class and 1518 belong to the negative class, and a test set with a total of 1130 variants, of which 476 belong to the positive class and 654 belong to the negative class. The final dataset is summarized in Table 2.

A mock-up illustrating the preprocessed data format is shown in Table 3.

#### Missingness

**Figure 3.**
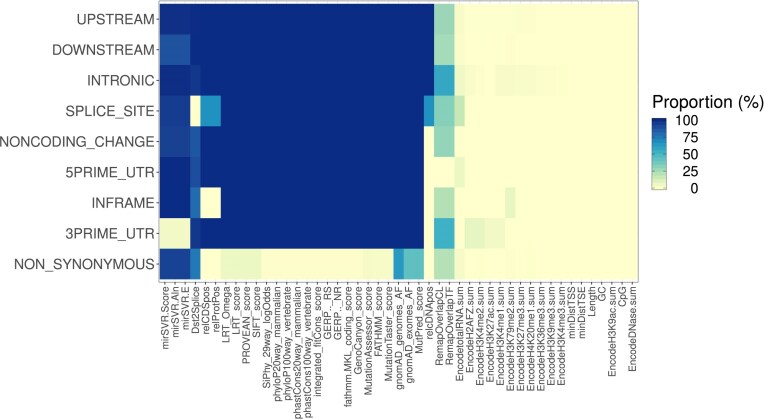
Missingness clusters based on predicted molecular consequence in the training set. Each cell displays the proportion of missing values of the indicated feature (horizontal axis) in the predicted molecular consequence class (vertical axis).

Missingness percentages of the features conditional on the VEP-predicted variant or transcript consequence are presented in Fig. 3. As expected, INTRONIC, UPSTREAM, DOWNSTREAM, NON-CODING_CHANGE, and 5PRIME_UTR variants exhibit large missingness percentages across protein-related features and microRNA (miRNA) predictions. Available features in these variants describe regulation. INFRAME variants have similar pattern, but have observed values in features describing variant position in coding sequence and protein codon. Such features are partially observed in SPLICE_SITE variants, possibly due to some of them also being interpretable as coding variants. SPLICE_SITE variants have the distance to a splice site completely observed. NON-SYNONYMOUS variants have nearly completely observed feature vectors, except for mostly unobserved miRNA downregulation scores [50] and incompletely observed gnomAD allele frequencies, MutPred predictions and REMAP2 [51] annotations.

Many features are only applicable to specific consequences, leading to few complete cases. The complete cases are the very few variants that have both splicing predictions as well as the features available for non-synonymous variants.

To assess whether there is informative missingness in the data, we computed each feature’s missingness indicator’s correlation to the outcome indicator (see Supplementary Fig. S10). Some correlation is present for most features. Also, most correlations are negative, implying lower likelihood of pathogenicity when the feature is missing. However, correlations for EncodetotalRNA.sum, gnomAD_exomes_AF, and gnomAD_genomes_AF are positive, implying that their missingness is linked to higher likelihood of pathogenicity.

### Included missingness handling methods

We include, in total, 14 missingness handling methods, consisting of six simple imputation methods, specificially mean, median, minimum, maximum, and zero imputation, four multiple imputation by chained equations (MICE) methods [52–54], three other popular imputation methods, specifically k-NN, BPCA [55], and missForest [56], and finally missingness indicator augmentation (see Table 4).

For k-NN, you must have enough complete cases to start imputation, depending on *k*. As the data had very few complete cases, the largest *k* that could be used was 2.

In the case of missingness indicators, features with identical missingness patterns produce identical indicator vectors, of which only one is kept.

Implementations of imputation methods often do not provide an easy way to reuse learned parameters from an earlier run. This makes it difficult to use parameters from the training set on the test set. We implement learned parameter use for simple imputation methods, and k-NN supports this directly in the DMwR package [58]. For simplicity of implementation, we choose to ignore the issue when out-of-the-box parameter reuse is not available. There is a possibility that this will give an advantage to simple methods and k-NN even if MICE, BPCA, and MissForest would otherwise outperform them. However, this comparison still represents the situation as it presents itself to the practitioner that may not have the time or expertise to make use of the other options.

The package mlr [60] offers wrapper functionality that allows use of any prediction method offered by the package also for univariate imputation, along with functionality for correct reimputing data with previously learned parameters. Using this option is difficult in a dataset with very few complete cases to use as training data, and as such we did not explore this possibility in this work. Investigation of the imputation performance of methods originally intended for prediction might merit further study.

### Experiments

#### Additional missingness experiment

We performed an experiment with additional, randomly generated missing values in the data in order to study

- the effect of missingness level to imputation method performance,
- the relation of an imputation method’s predictive performance to classifier performance.

Many imputation studies specifically assess an imputation method’s capability to predict the underlying values of a dataset with missing data, and use the root-mean-square error (RMSE) as a metric of performance of the imputation method. However, an imputation method with the lowest RMSE is not in general the best one in the statistical inference context: Van Buuren [29, chapter 2.6] uses the example of single imputation using a linear model fitted using least squares and thus minimizing RMSE. Analysis using single imputed data with minimal RMSE would still fail to account for the uncertainty from missing values, which would be correctly handled by multiple imputation (see the ‘Missingness handling in prediction’ section).

It is unclear whether RMSE remains a useful metric in imputing data for prediction. To investigate whether low RMSE on the training set predicts high classifier performance on the test set, we compute the RMSE of each imputation method on each dataset with additional missing values.

In many studies missing values are generated either on fully artificial data, or on the complete subsets of real datasets. However, there are very few complete cases in our dataset.

Thus, we chose to instead take the full, incomplete dataset as the basis of the experiment, and then used the following strategy:

- Induce varying amounts of missing values using ampute [61] on the original dataset
- Impute each generated dataset with each imputation method
- Compute RMSE for generated missing values against the original observed value
- Train a classifier on each imputed training dataset, and evaluate performance on imputed original test set

We generate 100 datasets for each of nine percentage categories of additional missingness, ranging from 10% to 90%. We therefore execute the framework on 900 datasets in total. Since MissForest is significantly more computationally expensive than other imputation methods, it is not included in the additional missingness experiment.

We impute each generated dataset using a sparser hyperparameter grid (downsampled to eight hyperparameter configurations separately for each dataset) and producing only one dataset each when using multiple imputation methods.

A classifier of each type is trained on the imputed datasets, the best performing imputation hyperparameter configuration is chosen by the highest performing classifier trained on a dataset imputed via that configuration.

Due to the removal of features with fewer than 1% unique values and features that highly correlate with another feature, the addition of missing values may lead to differing feature sets between different datasets with additional missingness. Especially large numbers of additional (MCAR) missing values may lead to fewer unique values, and both increase or decrease correlation between features by chance.

#### Cross-validation experiment

In the cross-validation experiment, we use repeated random sub-sampling with a 70% split over the training data to produce 100 training/test dataset pairs. The framework is run separately on each pair, after which the results can be used to estimate imputation methods’ relative robustness to variation from sampling. For speed, hyperparameters for imputation methods are downsampled to eight hyperparameter configurations each, and multiple imputation methods are set to produce only a single dataset. However, due to the fewer required executions of the framework (when compared to the additional missingness experiment), it was feasible to also include MissForest in the set of methods.

#### Main experiment

In the main experiment, the framework is run once with the full hyperparameter grid for each imputation method on the full training and test datasets, and MissForest is included in the set of methods. We also utilize the capability of multiple imputation methods and MissForest to produce multiple datasets to estimate the performance variability that arises from randomness in the imputation of both training and test sets using such methods. The methods are used to produce 10 imputations of both the training and the test sets. For each completed dataset, we train a separate classifier (performing its usual hyperparameter search and model selection procedure separately on each dataset).

Note that dynamic removal of features after splitting data to training and test sets may lead to duplicated feature vectors between training and test sets, i.e. a variant in the training set may end up sharing its feature vector with a different variant in the test set. We checked this for the main experiment and discovered 4 test set variants that became identical with a training set variant with the final feature set. Additionally, 2 variants had ended up with duplicated feature vectors with another variant within the training set, and 1 variant had been similarly duplicated within the test set. We removed all such duplicated variants from the test set in the main experiment.

### Metrics

We use *Matthews’ correlation coefficient* (MCC) as our main evaluation metric since it is less misleading than other common classification performance metrics in imbalanced datasets [62].

MCC is defined as


\begin{eqnarray*}
{\rm MCC} = \frac{{\rm TP} \times {\rm TN} - {\rm FP} \times {\rm FN}}{\sqrt{({\rm TP+FN})({\rm TP+FP})({\rm TN+FP})({\rm TN+FN})}}.
\end{eqnarray*}

We also present results with the *area under receiver operating characteristic curve* (AUC-ROC, or just AUC) metric, defined as the area under the receiver operating characteristic curve.

Finally, we compute the RMSE to compare imputed and original values in the additional missingness experiment. RMSE is defined as


\begin{eqnarray*}
\sqrt{\frac{1}{N} \sum _{i = 1}^{N}(\hat{y}_i - y_i)^2 }
\end{eqnarray*}

where *y*_*i*_ is the *i*th true value, $\hat{y}_i$ is the *i*th imputed value and *N* is the number of imputed values.

### Implementation

The framework and experiments were implemented with R [41] and organized into 11 executable scripts, presented in Table 6.

**Table 6.** Available scripts and their descriptions

Number	Description	File name
1.	Parsing of VCF file	01_parse_vcf.R
2.	Preprocessing	02_preprocess_data.R
3.	Computation of descriptive statistics	03_descriptive_stats.R
4.	Execution of training set imputation and classifier training for main experiment	04_run_impute_and_train.R
5.	Execution of test set imputation and prediction for main experiment	05_run_prediction.R
6.	Generation of datasets with additional missing values	06_generate_simulated_data.R
7.	Execution of imputation and classifier training on datasets with additional missing values	07_run_simulations.R
8.	Process and plot test set performance statistics for main experiment	08_analyze_results.R
9.	Process test set performance statistics for classifiers trained on datasets with additional missing values	09_analyze_simulation_results.R
10.	Plot test set performance statistics for classifiers trained on datasets with additional missing values	10_simulations_plots.R
11.	Perform repeated random sub-sampling cross-validation and process and plot its results	11_crossvalidation.R

The scripts are intended to be executed in order, but users may choose only run a subset if they are only interested in a subset of the results. To run the main experiment, one must run 1., 2., 4., 5., and 8.; to run the additional missingness experiment, one must run 1., 2., 6., 7., 9., and 10.; to run the cross-validation experiment, one must run 1., 2., and 11.

### Availability of source code and requirements

Project name: AMISS

Project home page: https://github.com/blueprint-genetics/amiss

Operating system(s): Linux, MacOS, Windows

Programming language: R [41]

Other requirements: The software was run with R 3.6.0 with packages vcfR [63], futile.logger [64], tidyr [65], here [66], magrittr [67], ggcorrplot [68], mice [54], foreach [69], doParallel [70], ggplot2 [71], iterators [72], missForest [56, 59], DMwR [58], doRNG [73], rngtools [74], lattice [75], itertools [76], randomForest [45], ModelMetrics [77], stringr [78], gridExtra [79], digest [80], purrr [81], caret [46, 82], testthat [83] and e1071 [84], and pcaMethods [57] via BioConductor [85] and BiocManager [86].

DMwR has since been removed from CRAN and k-NN imputation now depends on multiUS [87] in the repository. xgboost [88], ranger [89] and rjson [90] have been added as dependencies during further development.

License: MIT

Any restrictions to use by non-academics: CADD annotations require commercial users to contact authors for licensing. dbNSFP [49] annotations may require licenses for commercial use and must be reviewed individually.

## Results

This section is arranged as follows. First, we present results from the additional missingness experiment. We evaluate both the relation between classifier performance and RMSE, and how increasing missingness percentage affects classifier performance. Next, we present results from the repeated random sub-sampling cross-validation experiment, to evaluate the robustness of missingness handling methods to dataset composition. We then present results from the main experiment, comparing the classifier performances between missingness handling methods. Finally, we compare the missingness handling methods with respect to their running times.

### Additional missingness experiment

#### RMSE

Supplementary Figures S3–S6 plot RMSE against MCC for each imputation method. We found that RMSE and classification performance as measured by MCC did not correlate in datasets with additional missing values. This is especially clear in the case of outlier imputation, where RMSE is–as expected–much higher than with any other method, while MCC was comparable to other methods.

#### Missingness percentage

**Figure 4.**
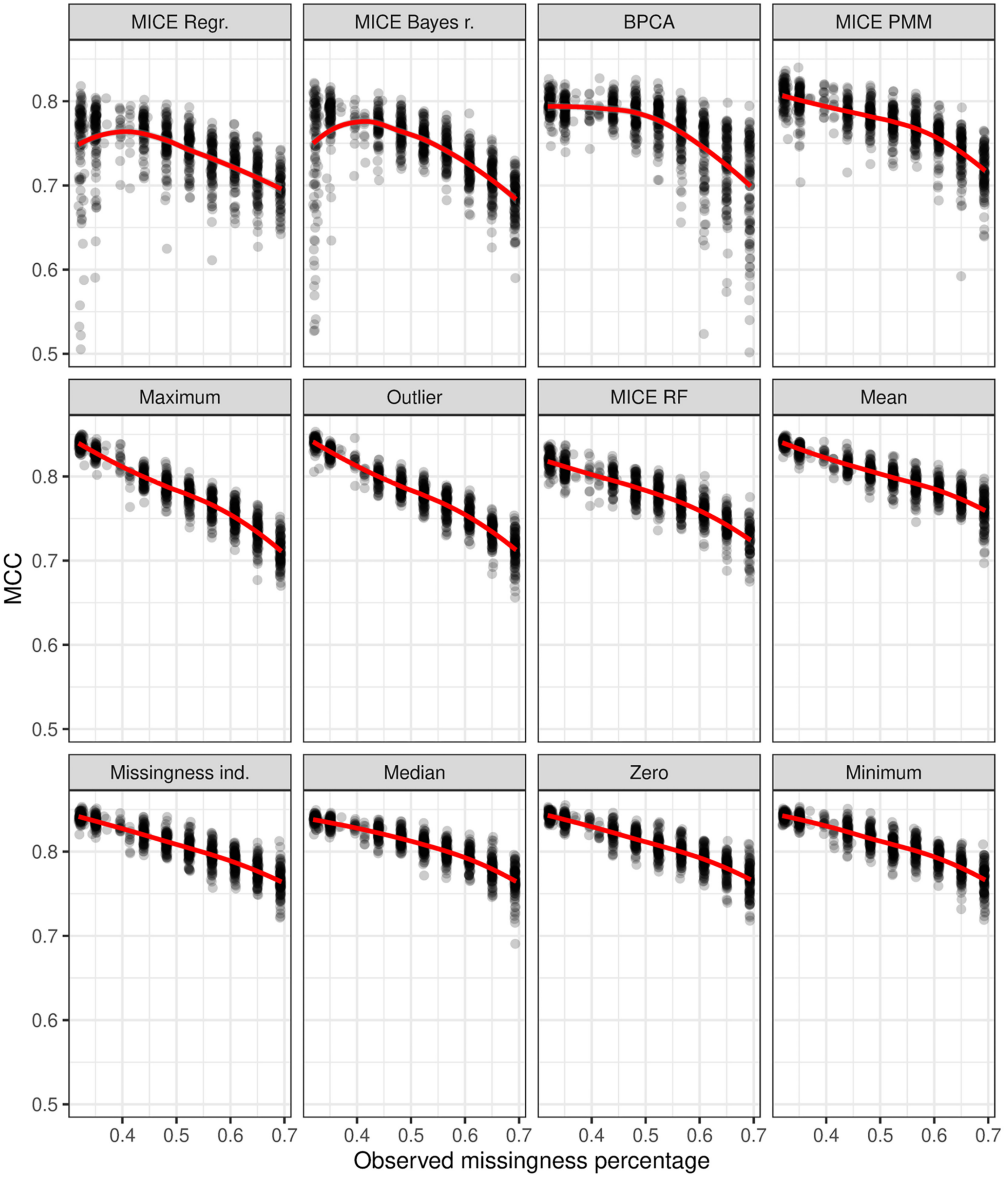
RF MCC on the test set against observed missingness percentage in the additional missingness experiment, with fitted LOESS curves. In the experiment, additional missing values are randomly generated to produce 100 datasets each for nine levels of additional missingness. Each imputation method is used on each dataset and classifiers are trained on the resulting complete datasets, and tested on the original test set completed by the same imputation method. The horizontal axis represents the observed missingness percentage, which includes both original and additional missingness. The vertical axis represents the test-set performance of the classifier.

The effect of additional MCAR missingness on MCC performance of classifiers is displayed in Fig. 4 and Supplementary Fig. S1. Fitted LOESS (locally estimated scatterplot smoothing) curves are shown.

When the classifier is a RF, missingness indicator augmentation as well as mean, minimum, zero and median imputations show similar curves, with their average performances dropping from slightly below MCC = 0.85 at 30% missingness to slightly above MCC = 0.75 at 70% missingness. MICE RF and MICE PMM have overall slightly lower mean performance than the previously mentioned methods, but otherwise show similar shapes. Outlier and maximum imputations suffer more drastically, with mean performances dropping to just above MCC = 0.70 at 70% missingness. MICE regression and MICE Bayes regression demonstrate a curious effect where average classifier performance increases at first as missingness increases, before starting their descent. BPCA shows hints of a similar but more muted trend. This may be related to a phenomenon noted by Poulos & Valle [91] in the context of prediction on categorical variables, where introduction of additional missing values prior to imputation may improve classifier performance.

When the classifier is LR, outlier imputation shows a dramatic drop immediately between 30% and 40% missingness and stabilizes slightly above MCC = 0.20. Maximum imputation shows a clear linear downward trend, while minimum imputation, zero imputation, and BPCA show a much smaller one. Missingness augmentation and all MICE methods show very light reductions in average performance as missing value percentage grows. Median and especially mean imputation show practically no performance reduction due to increasing MCAR missingness. MICE Bayes regression, BPCA, and PMM and especially MICE regression all show much larger variability in their performance than other methods.

### Cross-validation experiment

**Figure 5.**
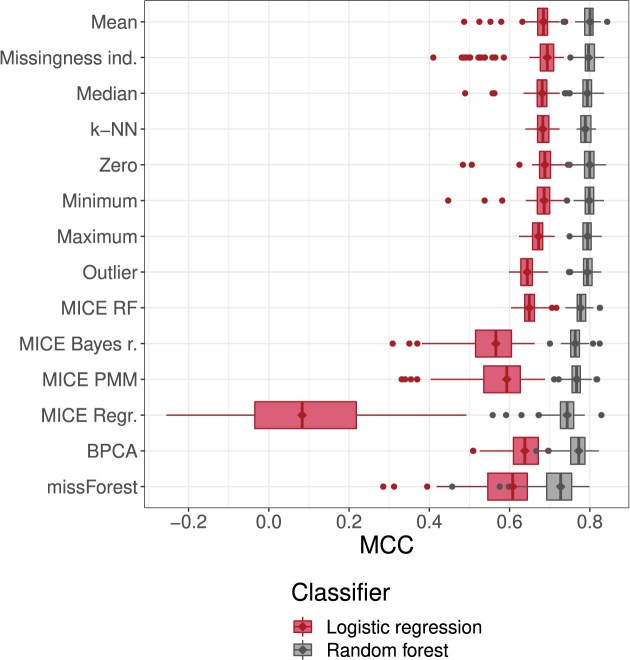
Classifier performance with respect to MCC measured by repeated random sub-sampling cross-validation in the ‘Cross-validation experiment’ section. In the experiment, the training set was randomly split 100 times into 70%/30% subsets. For each of the resulting pairs and each imputation method, both sets were imputed using the same method, and a classifier was trained on the larger subset and its performance was estimated using the smaller subset. Performance on the smaller subset is presented with boxplots (horizontal axis) per imputation method (vertical axis). Diamonds were added to emphasize median values.

The variability of classifier performance evaluated via repeated random sub-sampling cross-validation is displayed in Fig. 5.

The RF classifier outperforms LR regardless of imputation method, and the lowest performing imputation method wrt RF classifiers (MissForest) has higher mean performance than the highest performing imputation method wrt LR (missingness indicators).

For both LR and RF, single imputation methods and k-NN appear to have equivalent performance, though in conjunction with LR outlier imputation seems to perform slightly worse. With regard to RF classifiers, MICE RF, BPCA, MICE Bayes regression, and MICE PMM perform slightly or somewhat worse than simple imputation methods, but display greater differences in conjunction with LR, where MICE RF and BPCA are clearly preferable to MICE Bayes regression and MICE PMM. MICE ordinary regression and MissForest perform worse on average with both classifier types, but when combined with LR, MICE ordinary regression brings mean classification performance down close to that of a coin flip.

### Main experiment

In order to assess the differences in classifier performance due to selection of imputation method, we computed mean performance metrics for classifiers trained on datasets produced by each missingness handling method, sorted by MCC, for RF classifiers (Table 7) and for LR classifiers (Supplementary Table S1). Single imputation methods (mean, missingness indicator, median, k-NN, zero, minimum, maximum, outlier) produced only a single dataset, and as such also produced only a single set of performance statistics. MICE ordinary regression imputation was unable to produce imputations in the main experiment, and is thus excluded.

**Table 7.** Mean test set performance metrics for a RF classifier trained and tested on data sets treated with each missingness handling method

Method	MCC	AUC-ROC	Sensitivity	Specificity	*F*_1_	Precision
Maximum	0.847	0.974	0.911	0.937	0.912	0.913
Missingness ind.	0.845	0.975	0.907	0.937	0.910	0.913
k-NN imputation	0.844	0.972	0.918	0.929	0.911	0.904
Mean	0.843	0.974	0.913	0.934	0.911	0.909
Zero	0.839	0.975	0.905	0.933	0.906	0.907
Median	0.838	0.975	0.915	0.925	0.907	0.898
Minimum	0.838	0.975	0.903	0.934	0.906	0.909
Outlier	0.836	0.973	0.907	0.929	0.905	0.903
MICE PMM	0.814	0.975	0.867	0.940	0.889	0.914
MICE Bayes regr.	0.812	0.975	0.879	0.929	0.890	0.901
MICE RF	0.811	0.975	0.878	0.930	0.889	0.901
BPCA	0.781	0.974	0.763	0.982	0.853	0.968
MICE regr.	0.775	0.964	0.788	0.960	0.854	0.937
MissForest	0.759	0.956	0.818	0.930	0.854	0.895

In the case of RF classification, maximum imputation has the best average performance with respect to MCC (= 0.847). It is very closely followed by missingness indicator augmentation, k-NN imputation, and mean imputation, and then zero, median, minimum, and outlier imputation methods. MICE predictive mean matching is the highest performing MICE method (MCC = 0.814), with slightly better MCC than Bayes regression and MICE RF. The final group, with distinctly worse classifier MCC, consists of BPCA (MCC = 0.781), MICE ordinary regression (MCC = 0.775), and MissForest (MCC = 0.759).

Mean classification performance is lower across the board for LR see Supplementary Table S1. Missingness indicator augmentation is the winner in this scenario with MCC = 0.699. k-NN imputation (MCC = 0.681) and BPCA (MCC = 0.676) receive second and third place, after which mean imputation, zero imputation, minimum imputation, maximum imputation, outlier, and median imputations have essentially equal performance (MCC ranging from 0.672 to 0.667). MissForest and most MICE methods (MCC between 0.650 and 0.596) perform noticeably worse. MICE ordinary regression leads to abysmal results (MCC = 0.133).

**Figure 6.**
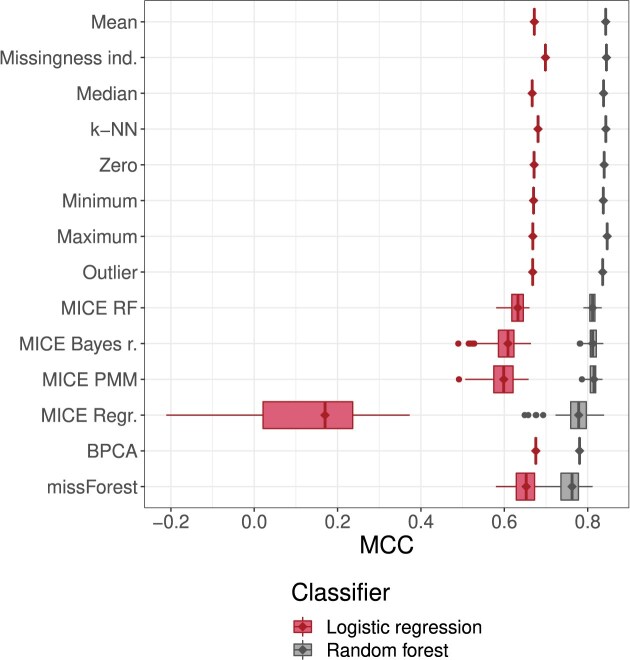
Classifier performance with respect to MCC on the test set in the ‘Main experiment’ section. In the experiment, each imputation method was executed on the full training set, classifiers were trained on the imputed dataset and the classifier’s performance was measured on the full test dataset imputed with the imputation method. Full hyperparameter grids for the imputation methods were used, and MissForest was included in the imputation methods. Classifier performance on the test set as measured with MCC is represented with boxplots (horizontal axis) per imputation method (vertical axis). Diamonds were added to emphasize median values. Variance, when present, is due to the production of 10 imputations of the same dataset by probabilistic or multiple imputation methods. A separate classifier is trained for each of the 10 imputed training sets, and each such classifier is evaluated on each of the 10 imputed test sets.

The MCC performance of classifiers is illustrated in Fig. 6. See Supplementary Fig. S2 for AUC-ROC performance. For multiple imputation and MissForest the variability of performance due to randomness in the imputation is also visible. Variability in classifier performance is seen to be smaller for RF compared to LR in all methods except for MissForest, and variability in general larger in methods with lower mean performance.

Using AUC-ROC to measure performance makes it more difficult to compare imputation methods due to the very small absolute differences. For a RF classifier (Table 7), the mean AUC-ROC of all methods, with the exception of MissForest and MICE ordinary regression, is within 0.003 of each other. For LR (Supplementary Table S1), the range is wider, and is also visually distinguishable in Supplementary Fig. S2. Interestingly, here MissForest has the upper hand. However, looking back at Supplementary Table S1 we notice that MissForest shows the highest specificity but also the lowest sensitivity of all methods. The imbalance is reflected in the relatively poor MCC and *F*_1_ scores, but ignored by AUC-ROC.

The results are not very different from the cross-validation experiment results in Fig. 5. When compared using MCC, simple imputation methods are largely interchangeable, though missingness indicators dominate when using a LR classifier, and maximum imputation is dominant when using a RF classifier. However, when considering the variance shown in the cross-validation experiment, the differences between simple imputation methods are likely to be due to chance. It is important to note that here the variance for multiple imputation methods (see Supplementary Tables S5 and S6 for standard deviations of performance metrics) is due to the multiple imputation (or multiple runs of a probabilistic imputation method) of training and test sets, and thus is not directly comparable to that of the cross-validation experiments.

#### Results grouped by consequence

**Figure 7.**
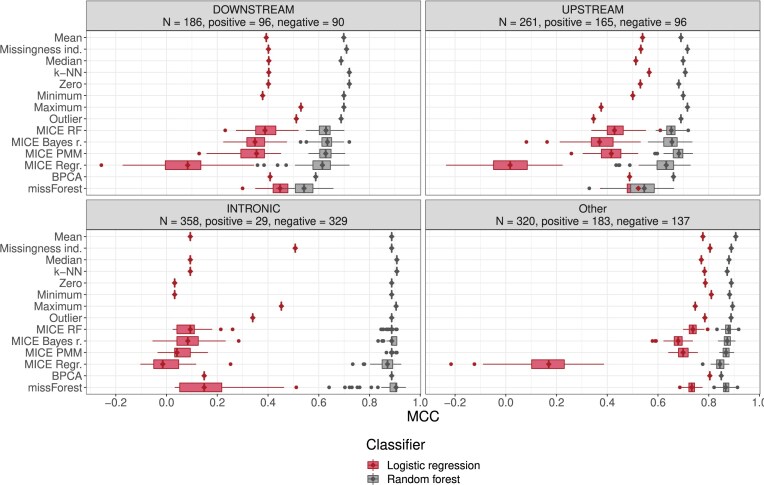
Classifier performance with respect to MCC in the the ‘Main experiment’ section, conditional on variant consequence. In the experiment, each imputation method was executed on the full training set, classifiers were trained on the imputed dataset and the classifier’s performance was measured on the full test set imputed with the imputation method. Full hyperparameter grids for the imputation methods were used, and MissForest was included in the imputation methods. Classifier performance on the test set as measured with MCC is represented with boxplots (horizontal axis) per imputation method (vertical axis), stratified by predicted variant consequence. Diamonds were added to emphasize median values. Variance, when present, is due to the production of 10 imputations of the same dataset by probabilistic or multiple imputation methods. A separate classifier is trained for each of the 10 imputed training sets, and each such classifier is evaluated on each of the 10 imputed test sets.

To assess whether the distinct missingness patterns exhibited within different variant consequence classes truly affect classification, we also computed performance statistics separately within certain consequence classes, specifically DOWNSTREAM, UPSTREAM, and INTRONIC, and finally Other, an aggregate of all remaining consequence classes. Other is formed by the consequence classes for which either (i) there are overall few variants, and the class indicator gets filtered out due to non-zero variance, or (ii) there is high correlation with another feature. Results are shown in Fig. 7.

Performances show large differences in different consequence classes. Other is mostly non-synonymous variants, as described above, and shows good performance for both classifier types, though RF is always superior. Mean imputation performs best with the RF classifier, while missingness indicator augmentation and minimum imputation maximize LR performance. The results are essentially equal to the general results depicted in Fig. 6 where consequence classes are not distinguished.

The consequence class INTRONIC shows a very different and interesting situation. First it is important to note that even after the preprocessing steps which removed variants in consequence classes with high class imbalance, only ∼8.1% of INTRONIC variants are positive, and we rely on the robustness of MCC to class imbalance allowing us to compare the performances. In this consequence class, LR is virtually useless, though missingness indicator augmentation and maximum imputation seem to allow some discriminatory power. It seems that in INTRONIC, LR learns to classify almost everything as negative (see Supplementary Figs S7 and S8). With most imputation methods, MCC is barely above zero. In contrast, the RF classifier performs well with any imputation method, though the high variation in MissForest imputations seems to allow also for instances of poor performance.

Compared to Other, prediction on variants of DOWNSTREAM consequence shows lower performance in both classifiers. For RF, the relative orders of imputation methods are basically the same as in the main experiment and the cross-validation experiment. Unlike in all other consequence classes, LR seems to perform better with MICE methods and MissForest than most simple imputation methods and k-NN, though maximum and outlier imputation do seem to outperform them even here.

In UPSTREAM variants, performance is again lower than in Other, but not as low as in DOWNSTREAM. RF classification shows the common pattern, where simple imputation methods and k-NN seem slightly preferable to MICE methods, and MissForest performs noticeably worse. LR classification looks, in this case, to be disadvantaged by the same methods that dominated DOWNSTREAM (that is, maximum and outlier imputation).

### Running time

Elapsed real time as well as CPU running time were recorded in the main experiment for the best hyperparameter configurations of all imputation methods and are presented in Table 8 and Supplementary Table S4, respectively. For methods that were used to produce multiple datasets (MICE methods and MissForest) the overall time was recorded and then divided by the number of produced datasets (10). The machine used to run the software was a CentOS Linux server with two Intel Xeon CPU E5-2650 v4 @ 2.20GHz processors and 512 GiB of memory. The analysis was run with parallelization using 24 processes. Each imputation was run inside a single process, and thus running times should not be affected based on whether an imputation method itself offers parallelization features.

**Table 8.** Running time for imputing the training set with the best hyperparameter configurations in the main experiment

Method	LR [elapsed (s)]	RF [elapsed (s)]
Zero	0.006	0.006
Maximum	0.007	0.007
Minimum	0.007	0.007
Mean	0.009	0.009
Median	0.011	0.011
Outlier	0.014	0.014
Missingness indicator augmentation	0.478	0.478
MICE Bayes regression	5.379	5.379
MICE regression	5.839	5.839
k-NN	12.792	12.948
BPCA	14.884	19.511
MICE PMM	15.427	14.921
MICE RF	107.036	104.278
MissForest	362.352	362.352

MICE regression and MICE Bayes regression show similar running times and finish between 5 and 6 s, while k-NN, MICE PMM, and BPCA take somewhat longer. MICE RF and especially MissForest take much longer. Simple imputation methods are much faster than all other methods.

## Discussion

RF classifiers outperformed LR overall, with the worst-performing combination of missingness handling method and RF still reaching higher performance than best-performing combination of missingness handling method and LR. This was expected, due to both the inherent capability of tree predictors such as those in RF to ignore irrelevant features, and the more flexible decision boundary that RFs are able to form. Choice of missingness handling method thus cannot compensate for an unsuitable classification method.

The high comparative performance of k-NN with both classifiers is surprising. The depletion of complete cases greatly limits the possible values for *k*. Even more importantly, the few complete cases are the only possible neighbors to any point; when *k* equals the number of complete cases, every missing value in a specific feature will be imputed with the average value of that feature in the set of complete cases. k-NN imputation with *k* equaling the number of complete cases can thus be interpreted to be a form of unconditional imputation, since in such a case every missing value will be imputed with a single value, irrespective of the values of other features.

The computational cost of different methods varies dramatically. Many of the highest-performing methods take a minuscule portion of time spent during the overall training and prediction process, while more costly methods dominate the time requirements. The difference between zero imputation (0.006 s runtime on our dataset) to MissForest (362.352 s) may be inconsequential for a single sample, but is compounded with large cohorts or high-throughput diagnostic work. For example, for 100 000 WGS samples the difference in total computational time with the simplest and most complex method will be over a year, potentially translating into hundreds of thousands of euros of additional cloud computing and storage costs.

Simple imputation methods overall could be considered the winning group of the comparison presented in this paper, and mean imputation might further be pointed out as the favorite in that group. In addition to the consistently high MCC in all experiments, it also displays high resilience to performance degradation due to increasing MCAR missingness, and low variance in relation to sampling. It is also one of the fastest methods to compute, trivial to implement and is always applicable.

Considering the complexity of the prediction task, the features, the large degree of missingness, and the sophistication of available missingness handling strategies, it is somewhat surprising that the best performance is gained using simple unconditional imputation strategies.

In contrast, Perez-Lebel *et al.* [92] find that for predicting on health databases, supervised learning methods with built-in missingness handling perform best. We did not include built-in missingness handling in our analysis, so results are not directly comparable. However, they also find that constant imputation with additional missingness indicator variables perform well.

### Effect of missingness mechanism

Though the we only induce MCAR missingness in the simulations, it is very likely that MAR and MNAR missingness is present in the data, as the data consists of real-world variants and their real annotations. MAR and MNAR mechanisms are thus not ignored by the experiments, though their effects are difficult to isolate.

Still, we can attempt to a priori analyze the effect of missingness mechanism on downstream classifiers.

A missingness mechanism on one feature that is dependent on the observed values of another is classified as MAR. Any correlation of the observed values of the second feature to the response variable is equal to the informativeness of the first feature. Any information used to impute the underlying values beneath missingness generated by the missingness mechanism is also already available to the classifier to predict the response without imputation. When a missingness mechanism is (at least partially) dependent on unobserved values, then the informativeness of the feature with missingness is still available to predict the response, but less information is available to impute the feature itself. At worst, imputation may confound the classifier by producing values that only by chance correlate with the response. Any correlation of the underlying values of the feature with missingness to the response is unavailable to the classifier.

This suggests that informativeness is a key property of features for prediction, and it is essential that the classifier is able to distinguish imputed values in order to make use of informativeness. Our results corroborate this, as missingness indicators and single imputation methods obtain the best downstream classifier performance.

### Informativeness

Indeed, informativeness may be one major factor to the observed rankings between imputation methods.

If the presence of a missing value in certain variables correlates with the pathogenicity of the variant, simple imputation methods would be given an advantage: when every imputed value (within a given feature) is replaced by a single specific value, the classifier may learn to correlate that single value with the outcome. This is less likely to happen when using more sophisticated imputation methods, which make it harder for the classifier to learn which values were likely imputed. However, LR is less flexible and thus less capable of representing such potentially discontinuous dependencies, and missingness indicator augmentation performs slightly better than most simple imputation methods when combined with LR. Further gains might be impeded by the dimensionality increase due to the additional indicator features.

#### Inability to transfer training parameters to test set

As described in the ‘Included missingness handling methods’ section, many available implementations of imputation methods do not allow reuse of parameters between imputation of training and test sets. Thus the estimated distributions on which imputed values are based will differ at least slightly, and thus disadvantage any methods for which parameter reuse was not possible.

However, the variability in imputations of the test set by stochastic and multiple imputation methods allows some estimation of the effect. If we assume that the performance gain from parameter reuse at best matches the upper range of performance variation on the test set, we see from Fig. 6 that MICE methods and MissForest at best match the performance of single imputation methods.

### Additional challenges

Grimm *et al.* [93] described several biasing factors in VEP training and performance evaluation using data from commonly used variant databases, e.g. the tendency for variants within the same gene being classified as all pathogenic or all neutral, or simply due to difficulty of finding datasets completely disjoint with the training set. Mahmood *et al.* [94] further analyzed existing VEPs using datasets generated from functional assays, and found drastically lower performance compared to earlier reported estimates.

Our approach is not immune to these biases, and we expect that any reported performance metrics will be overoptimistic. However, we expect that the main result of the study, the relative performance rankings of missingness handling methods, is not affected by the biases. The classifiers built described in this work are not intended to outperform earlier approaches or be directly used for variant effect prediction.

### Conclusions

It appears that it is unnecessary to use sophisticated missingness handling methods to treat missing values when building variant pathogenicity metapredictors. Instead, simple unconditional imputation methods and even zero imputation give higher performance and save significant computational time, leading to considerable cost savings if adopted. This highlights the conceptual separation between missingness handling methods for prediction and imputation for statistical inference, the latter of which requires carefully constructed techniques to reach correct conclusions.

### Further work

There are several ways to improve and expand on this work. The dataset could be extended to include variants from wider sources, and the effect of circularity could be estimated using additional datasets. It would likely also be possible to make changes to or reimplement methods whose implementation does not currently support reuse of parameters. Reduced models and its hybrid variants would make an interesting point of comparison if implemented. Another possible extension of the work would be to broaden the focus from missingness handling to various other design choices that may affect predictor performance, such as using random search in place of grid search or downsampling data to improve class balance. Extending simulations by allowing MAR, MNAR, or informative missingness and related experiments would also clarify the impact of missingness mechanism on imputation method choice.

## Potential implications

We compared a variety of commonly used missingness handling methods in order to assess their suitability in building ML based variant pathogenicity metapredictors. The analysis will help pathogenicity predictor developers choose missingness handling methods that maximize the performance of their tools, resulting in better accuracy, faster computation, and lower costs.

## Supplementary Material

lqaf133_Supplemental_File

## Data Availability

The result files from experiments described in this article are available in the Zenodo repository, DOI 10.5281/zenodo.6656616 [95].
