# Supplementary material for: Comparison of missing data handling methods for variant pathogenicity predictors
Source: NAR Genom Bioinform. 2025 Oct 14;7(4):lqaf133. doi: 10.1093/nargab/lqaf133 (PMC12526054; doi:10.1093/nargab/lqaf133)
Supplement: lqaf133_Supplemental_File [file lqaf133_supplemental_file.pdf]

Comparison of missing data handling methods for variant pathogenicity predictors

Mikko Särkkä[1,2], Sami Myöhänen[1], Kaloyan Marinov[1], Inka Saarinen[1], Leo Lahti[3], Vittorio Fortino[2], and Jussi Paananen[1,2]

[1]Blueprint Genetics Ltd, Helsinki, Finland
[2]University of Eastern Finland, Department of Health Sciences, School of Medicine, Institute of Biomedicine, Kuopio, Finland
[3]University of Turku, Faculty of Technology, Department of Computing, Turku, Finland

Supplementary information

Supplementary tables

Supplementary table S1 displays mean test-set performance of logistic regression classification with each missingness handling method.

Supplementary table S2 displays the frequencies of different predicted variant consequences in the full dataset (including both training and test datasets). Some consequence classes were very sparse, and the pre-imputation filtering process eliminated the dummy variables encoding class membership of `INFRAME`, `NONCODING_CHANGE`, `5PRIME_UTR`, `3PRIME_UTR`, and `SPLICE_SITE`. In addition, membership to class `NON_SYNONYMOUS` was strongly (negatively) correlated to the missingness of LRT predictions, and was also eliminated by the pre-imputation feature filtering. Thus these classes were grouped into `Other`.

Table S3 lists the features used in AMISS as well as their types and sources. For more authoritative descriptions of features, see the README file of dbNSFP3.5 found at https://sites.google.com/site/jpopgen/dbNSFP and supplementary table S2 of Rentzsch P, Witten D, Cooper GM, Shendure J, Kircher M. CADD: predicting the deleteriousness of variants throughout the human genome. Nucleic Acids Res. 2019 Jan 8;47(D1):D886-D894.

Table S4 presents the CPU time spent on imputing the training set by each imputation method with the hyperparameter configurations with the highest-performing downstream random forest and logistic regression classifiers, respectively.

Table S5 presents the standard deviations of performance metrics of random forest classifiers on the test set in the Main experiment.

Table S6 presents the standard deviations of performance metrics of random forest classifiers on the test set in the Main experiment.

Supplementary figures

Figure S1 shows logistic regression performance on the test set as a function of missingness percentage. Each subplot represents the performance of a set of classifiers trained on 900 datasets with additional

Supplementary Table S1: Main experiment: Mean test-set performance metrics for a logistic regression classifier trained and tested on data sets treated with each missingness handling method

Method	MCC	AUC-ROC	Sensitivity	Specificity	F_1	Precision
Missingness ind.	0.699	0.924	0.839	0.862	0.827	0.815
k-NN imputation	0.681	0.923	0.816	0.865	0.815	0.814
BPCA	0.676	0.922	0.831	0.848	0.815	0.799
Mean	0.672	0.919	0.814	0.859	0.811	0.807
Zero	0.672	0.923	0.808	0.863	0.809	0.811
Minimum	0.670	0.921	0.812	0.859	0.809	0.807
Maximum	0.668	0.913	0.827	0.845	0.810	0.795
Outlier	0.668	0.906	0.863	0.813	0.814	0.770
Median	0.667	0.920	0.814	0.854	0.808	0.802
MissForest	0.650	0.931	0.697	0.924	0.772	0.872
MICE RF	0.632	0.906	0.740	0.881	0.777	0.818
MICE Bayes regr.	0.600	0.885	0.786	0.814	0.768	0.758
MICE PMM	0.596	0.896	0.696	0.880	0.744	0.814
MICE regr.	0.133	0.598	0.526	0.592	0.483	0.533

Supplementary Table S2: Numbers of occurrence of variant consequence classes.

Consequence	Occurrences
`INFRAME`	14
`NONCODING_CHANGE`	14
`5PRIME_UTR`	18
`3PRIME_UTR`	27
`SPLICE_SITE`	53
`DOWNSTREAM`	464
`NON_SYNONYMOUS`	578
`UPSTREAM`	632
`INTRONIC`	806

Supplementary Table S3: Features used in AMISS.

Feature	Description	Source	Type
SIFT_score	Prediction from the SIFT insilico predictor	dbNSFP3.5	Numeric
LRT_score	Prediction from the LRT insilico predictor	dbNSFP3.5	Numeric
LRT_pred	3-category prediction from LRT	dbNSFP3.5	Categorical
LRT_Omega	LRT nonsynonymous to synonymous ratio	dbNSFP3.5	Numeric
MutationTaster_score	Prediction from MutationTaster	dbNSFP3.5	Numeric
MutationAssessor_score	Prediction from MutationAssessor	dbNSFP3.5	Numeric
FATHMM_score	Prediction form FATHMM	dbNSFP3.5	Numeric
PROVEAN_score	Prediction from PROVEAN	dbNSFP3.5	Numeric
MutPred_score	Prediction from MutPred	dbNSFP3.5	Numeric
fathmm.MKL_coding_score	Prediction from FATHMM MKL	dbNSFP3.5	Numeric
GenoCanyon_score	Prediction from GenoCanyon	dbNSFP3.5	Numeric
integrated_fitCons_score	fitCons conservation score	dbNSFP3.5	Numeric
GERP.._NR	GERP++ neutral rate	dbNSFP3.5	Numeric
GERP.._RS	GERP++ conservation score	dbNSFP3.5	Numeric
phyloP100way_vertebrate	phyloP100way vertebrate conservation score	dbNSFP3.5	Numeric
phyloP20way_mammalian	phyloP20way mammalian conservation score	dbNSFP3.5	Numeric
phastCons100way_vertebrate	phastCons100way vertebrate conservation score	dbNSFP3.5	Numeric
phastCons20way_mammalian	phastCons100way mammalian conservation score	dbNSFP3.5	Numeric
SiPhy_29way_logOdds	SiPhy 29 way log odds conservation score	dbNSFP3.5	Numeric
gnomAD_exomes_AF	gnomAD allele frequency from exomes	dbNSFP3.5	Numeric
gnomAD_genomes_AF	gnomAD allele frequency from genomes	dbNSFP3.5	Numeric
Length	Length of variant	CADD annot.	Numeric
GC	Percent of GC	CADD annot.	Numeric
CpG	Percent of CpG	CADD annot.	Numeric
relcDNApos	Relative position in transcript	CADD annot.	Numeric
relCDSpos	Relative position in coding sequence	CADD annot.	Numeric
relProtPos	Relative position in protein codon	CADD annot.	Numeric
Dst2Splice	Distance to splice site	CADD annot.	Numeric
minDistTSS	Distance to transcribed sequence start	CADD annot.	Numeric
minDistTSE	Distance to transcribed sequence end	CADD annot.	Numeric
mirSVR.Score	mirSVR score	CADD annot.	Numeric
mirSVR.E	mirSVR E	CADD annot.	Numeric
mirSVR.Aln	mirSVR Aln	CADD annot.	Numeric
EncodeH3K4me1.sum	Encode expression data	CADD annot.	Numeric
EncodeH3K4me2.sum	Encode expression data	CADD annot.	Numeric
EncodeH3K4me3.sum	Encode expression data	CADD annot.	Numeric
EncodeH3K9ac.sum	Encode expression data	CADD annot.	Numeric
EncodeH3K9me3.sum	Encode expression data	CADD annot.	Numeric
EncodeH3K27ac.sum	Encode expression data	CADD annot.	Numeric
EncodeH3K27me3.sum	Encode expression data	CADD annot.	Numeric
EncodeH3K36me3.sum	Encode expression data	CADD annot.	Numeric
EncodeH3K79me2.sum	Encode expression data	CADD annot.	Numeric
EncodeH4K20me1.sum	Encode expression data	CADD annot.	Numeric
EncodeH2AFZ.sum	Encode expression data	CADD annot.	Numeric
EncodeDNase.sum	Encode expression data	CADD annot.	Numeric
EncodetotalRNA.sum	Encode expression data	CADD annot.	Numeric
RemapOverlapTF	Remap binding TF number	CADD annot.	Numeric
RemapOverlapCL	Remap TF - cell line combinations	CADD annot.	Numeric
Dst2SplType	Type of splice site	CADD annot.	Categorical
Consequence.x	Molecular consequence prediction	VEP	Categorical

Supplementary Table S4: CPU time for imputing the training set with the best hyperparameter configurations in the main experiment.

Method	LR (s)	RF (s)
Zero	0.006	0.006
Maximum	0.007	0.007
Minimum	0.007	0.007
Mean	0.01	0.01
Median	0.012	0.012
Outlier	0.013	0.013
Missingness indicator augmentation	0.471	0.471
MICE Bayes regression	5.3577	5.3577
MICE regression	5.7866	5.7866
k-NN	11.556	11.548
BPCA	13.942	19.504
MICE PMM	15.3582	14.8809
MICE RF	106.2895	103.7417
MissForest	360.7571	360.7571

Supplementary Table S5: Standard deviations of performance statistics of random forest classifiers on the test set in the main experiment.

Method	*MCC*	*AUC*	*Sensitivity*	*Specificity*	*F*1	*Precision*
MICE regression	0.035	0.010	0.064	0.019	0.032	0.023
MissForest	0.027	0.010	0.034	0.018	0.018	0.021
MICE Bayes regr.	0.011	0.001	0.020	0.013	0.008	0.015
MICE PMM	0.011	0.002	0.017	0.013	0.007	0.016
MICE RF	0.009	0.001	0.013	0.008	0.006	0.010

Supplementary Table S6: Standard deviations of performance statistics of logistic regression classifiers on the test set in the main experiment.

Method	*MCC*	*AUC*	*Sensitivity*	*Specificity*	*F*1	*Precision*
MICE regression	0.141	0.109	0.225	0.288	0.106	0.107
MICE PMM	0.036	0.009	0.094	0.044	0.043	0.037
MICE Bayes regr.	0.034	0.015	0.078	0.051	0.029	0.038
MissForest	0.029	0.003	0.066	0.024	0.032	0.025
MICE RF	0.018	0.005	0.023	0.011	0.013	0.013

missing values, treated with the indicated missingness handling method. Each dot represents a performance value of a classifier on the original test set. MICE ordinary regression shows a markedly high variance and low mean performance. Mean and median imputation and missingness indicator augmentation both show highest performance when used with logistic regression as well. They also display little deterioration as MCAR missingness increases, suggesting that the classifier already learns most of its discriminative ability from a small amount of the available information. At the same time in manuscript figure 4. the deterioration is more pronounced for every missingness handling method, suggesting that random forest is able to utilize more subtle patterns in the data, and that those patterns are then more easily lost when additional missingness is simulated.

Figure S2 shows classifier performance in the Main experiment.

Figures S3 and S4 show classifier performance in the additional missingness experiment, plotted against RMSE across columns for a subset of the imputation methods, plotted with equal scales for each method. Outlier imputation is excluded here since its RMSE values are much higher than other methods. As can be seen in the figure, RMSE and MCC are not correlated, especially well illustrated by maximum, minimum and zero imputation, where high MCC is reached even as RMSE is consistently higher than for other methods.

Figures S5 and S6 are as above, except with only the MCC scale fixed. Outlier imputation and BPCA have drastically higher RMSE, but outlier imputation still shows good MCC values with a random forest classifier.

Figures S7 and S8 show the sensitivity and specificity, respectively, for test set variants of different consequence classes in the main experiment. Logistic regression seems to have little discriminatory power in `INTRONIC` variants when combined with most missingness handling methods, as sensitivity is near zero, implying all variants in this consequence class are being classified as benign. Missingness indicators, outlier imputation and maximum imputation seem to allow the method to better detect pathogenic variants, though still at a lower rate than random forest.

Figure S9 shows correlations in the training data from feature values to the positive outcome indicator.

Figure S10 shows correlations in the training data from the missingness indicators of each feature to the positive outcome indicator. Consequence.x, CpG, EncodeDNAse.sum, EncodeH3K9ac.sum, GC, Length, minDistTSE and minDistTSS have no missing values and thus have an empty correlation bar. For most features, the correlation is negative, implying that missingness in those features decreases the likelihood of pathogenicity. Correlations from missingness indicators of EncodetotalRNA.sum, gnomAD_exomes_AF and gnomAD_genomes_AF are positive, implying that missingness in those features increases the likelihood of pathogenicity.

Figures S11 through S18 show the histograms of feature values for each feature on the training set.

Figure S19 presents Pearson correlations between numeric features in the training set.

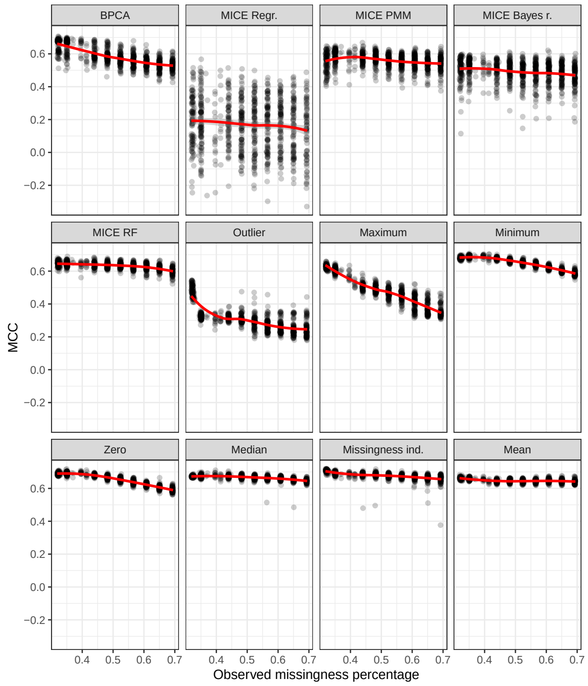

Supplementary Figure S1: Logistic regression MCC on the test set against observed missingness percentage in the additional missingness experiment, with fitted LOESS curves (red). In the experiment, additional missing values are simulated to produce 100 datasets each for nine levels of additional missingness. Each imputation method is used on each dataset and classifiers are trained on the resulting complete datasets, and tested on the original test set completed by the same imputation method. The horizontal axis represents the observed missingness percentage, which includes both original and simulated missingness. The vertical axis represents the test-set performance of the classifier.

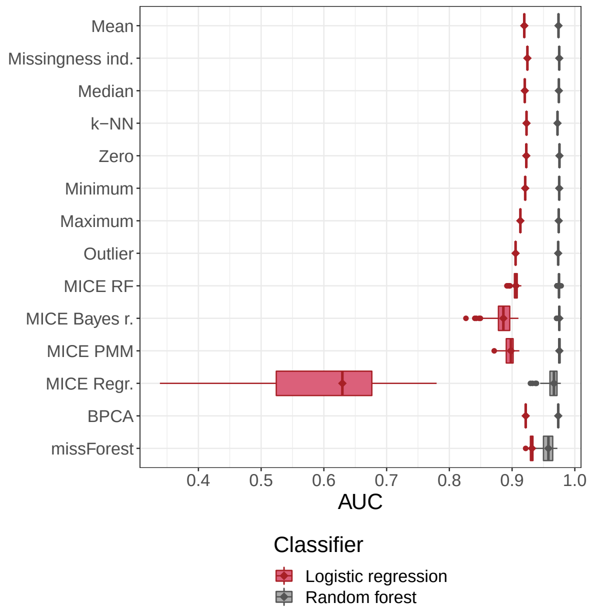

Supplementary Figure S2: Classifier performance with respect to AUC-ROC in the Main experiment. In the experiment, each imputation method was executed on the full training set, classifiers were trained on the imputed dataset and the classifier's performance was measured on the full test dataset imputed with the imputation method. Full hyperparameter grids for the imputation methods were used, and MissForest was included in the imputation methods. Classifier performance on the test set as measured with AUC-ROC is represented with boxplots (horizontal axis) per imputation method (vertical axis). Diamonds were added to emphasize median values. Variance, when present, is due to the production of 10 imputations of the same dataset by probabilistic or multiple imputation methods. A separate classifier is trained for each of the 10 imputed training sets, and each such classifier is evaluated on each of the 10 imputed test sets.

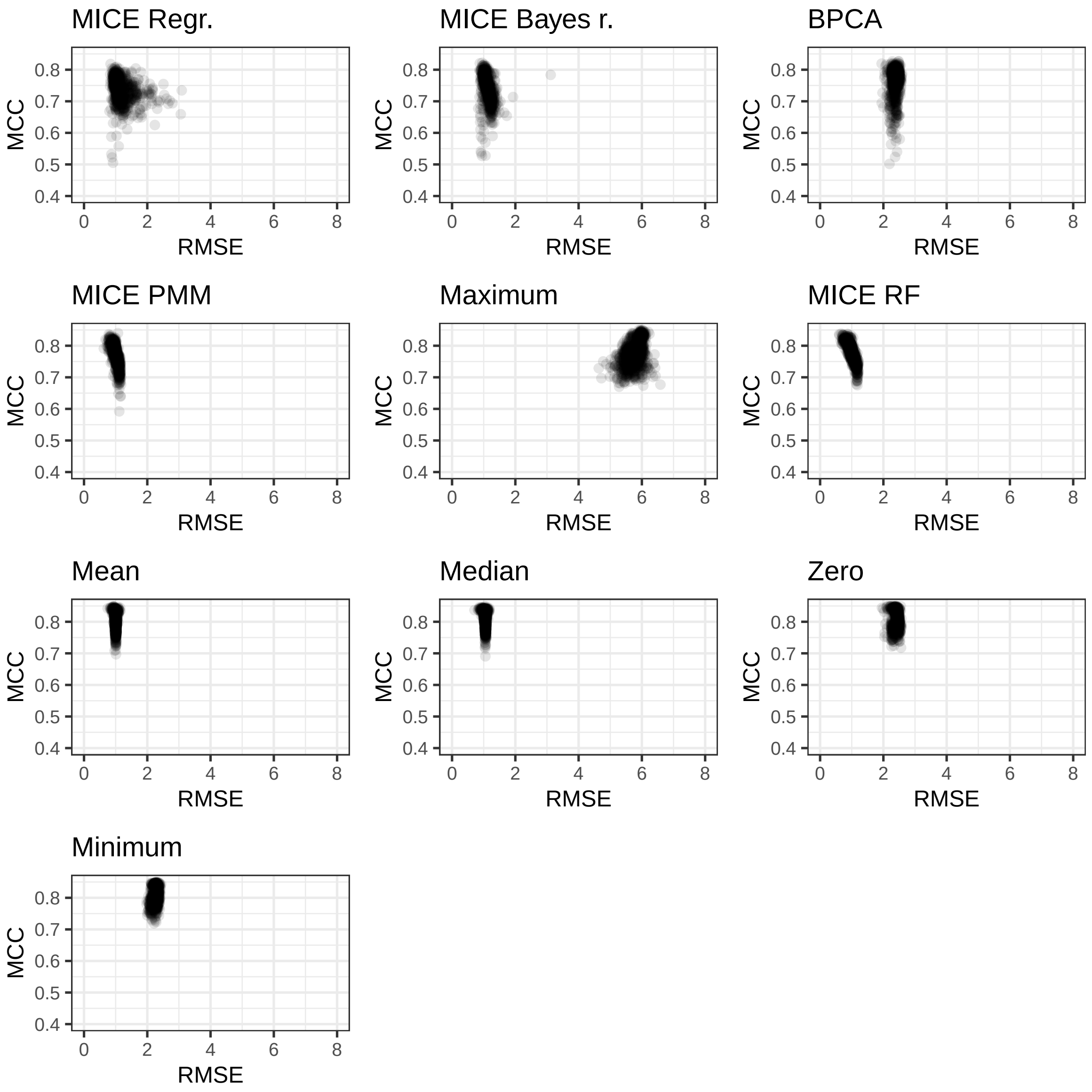

Supplementary Figure S3: Random forest performance against imputation error averaged across columns in the additional missingness experiment. Classifier performance is measured with MCC (vertical axis) and imputation error is measured with RMSE (horizontal axis). Subplots represent imputation methods used to complete the datasets with simulated additional missingness. All subplots use equal scales.

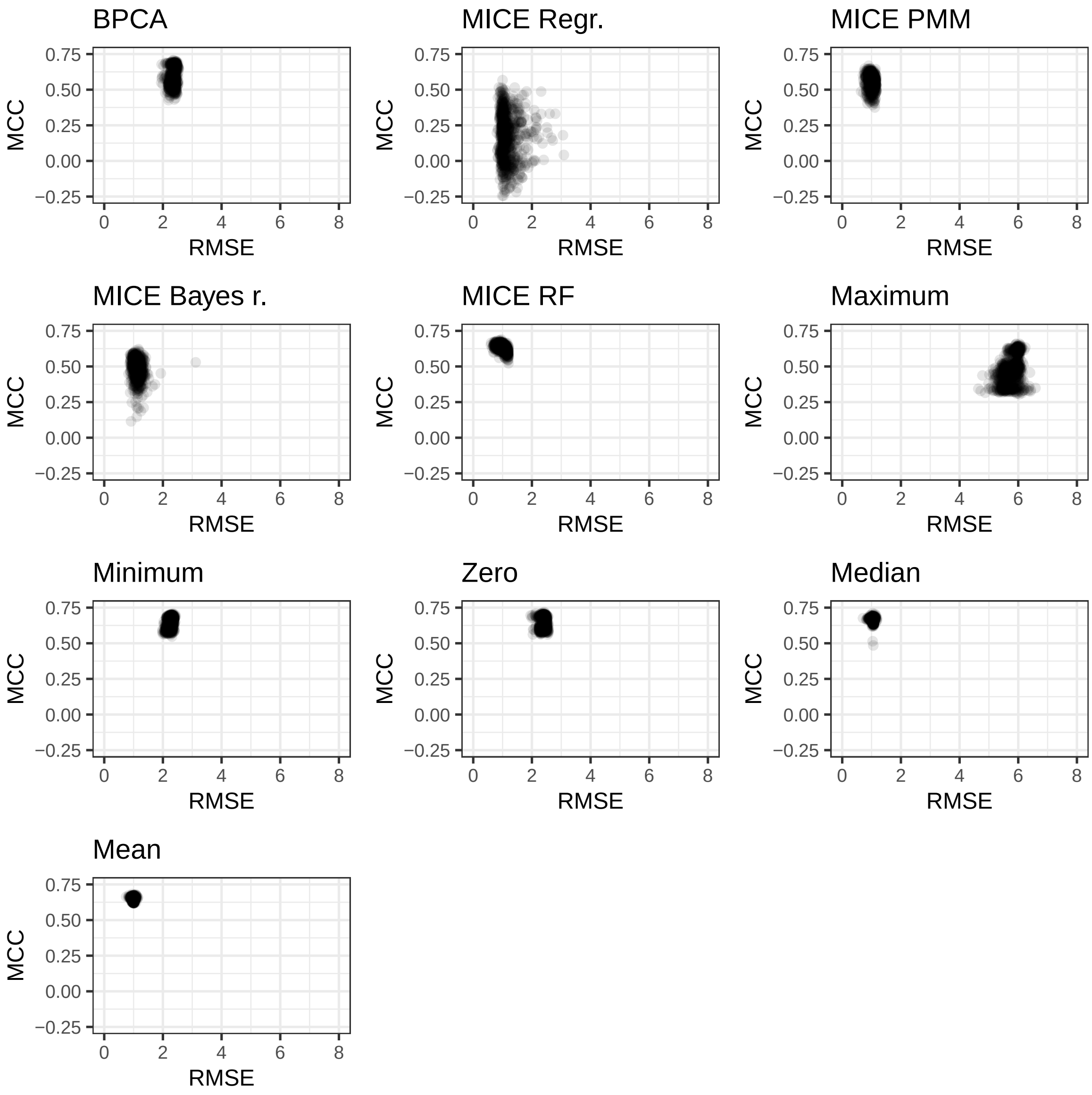

Supplementary Figure S4: Logistic regression performance against imputation error averaged across columns in the additional missingness experiment. Classifier performance is measured with MCC (vertical axis) and imputation error is measured with RMSE (horizontal axis). Subplots represent imputation methods used to complete the datasets with simulated additional missingness. All subplots use equal scales.

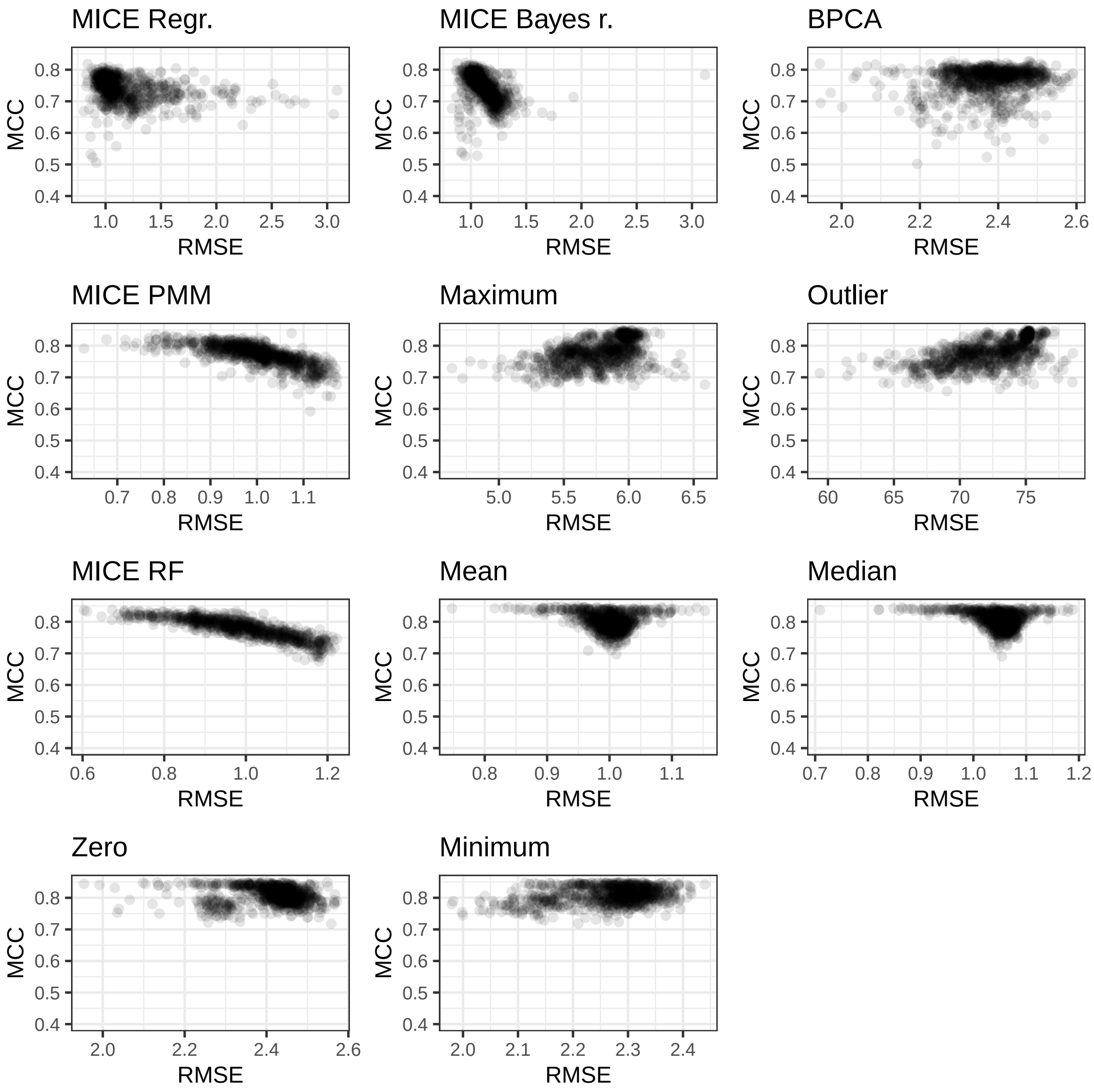

Supplementary Figure S5: Random forest performance against imputation error averaged across columns in the additional missingness experiment. Classifier performance is measured with MCC (vertical axis) and imputation error is measured with RMSE (horizontal axis). Subplots represent imputation methods used to complete the datasets with simulated additional missingness. The horizontal axis is scaled separately in individual subplots.

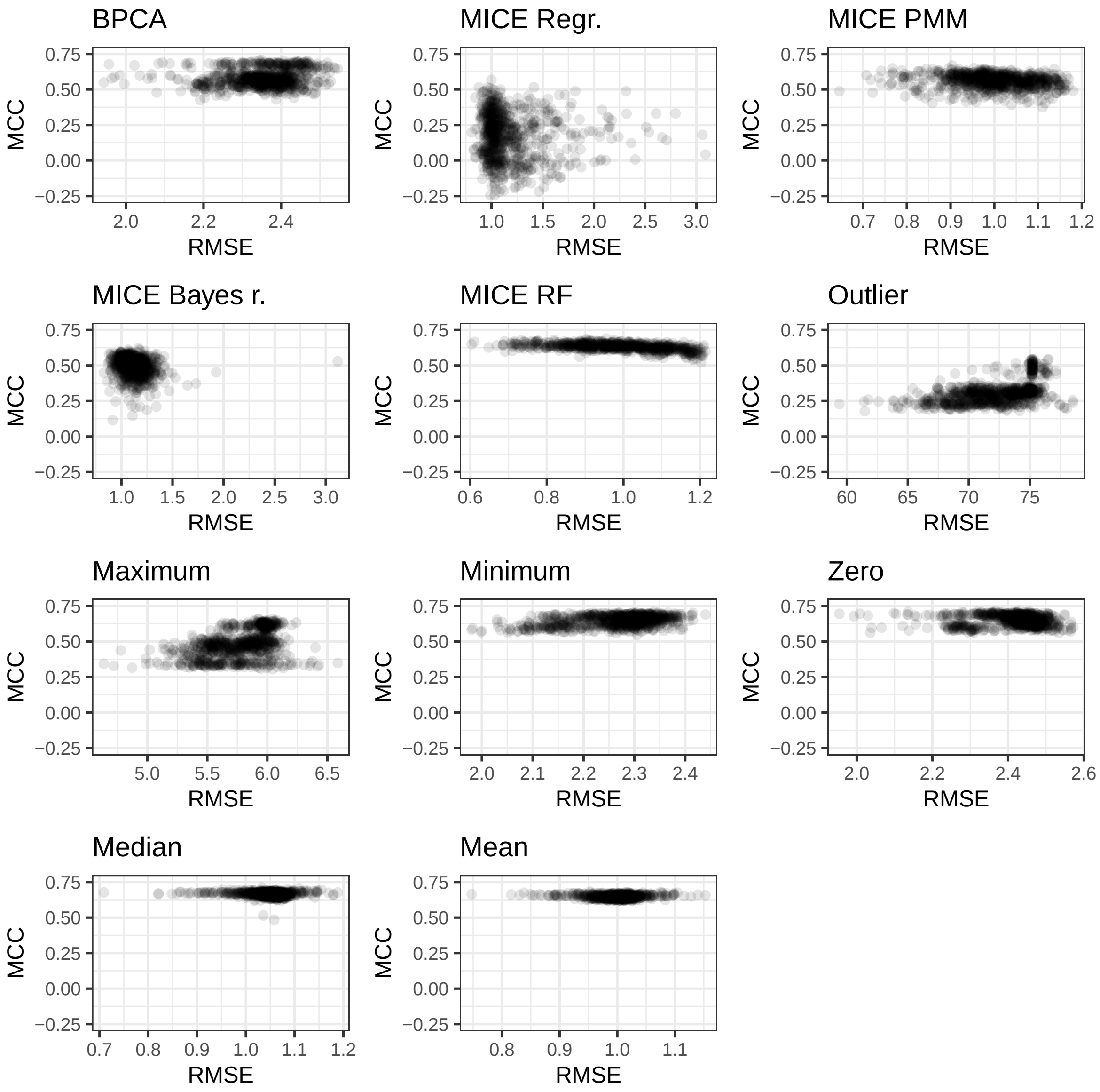

Supplementary Figure S6: Logistic regression performance against imputation error averaged across columns in the additional missingness experiment. Classifier performance is measured with MCC (vertical axis) and imputation error is measured with RMSE (horizontal axis). Subplots represent imputation methods used to complete the datasets with simulated additional missingness. The horizontal axis is scaled separately in individual subplots.

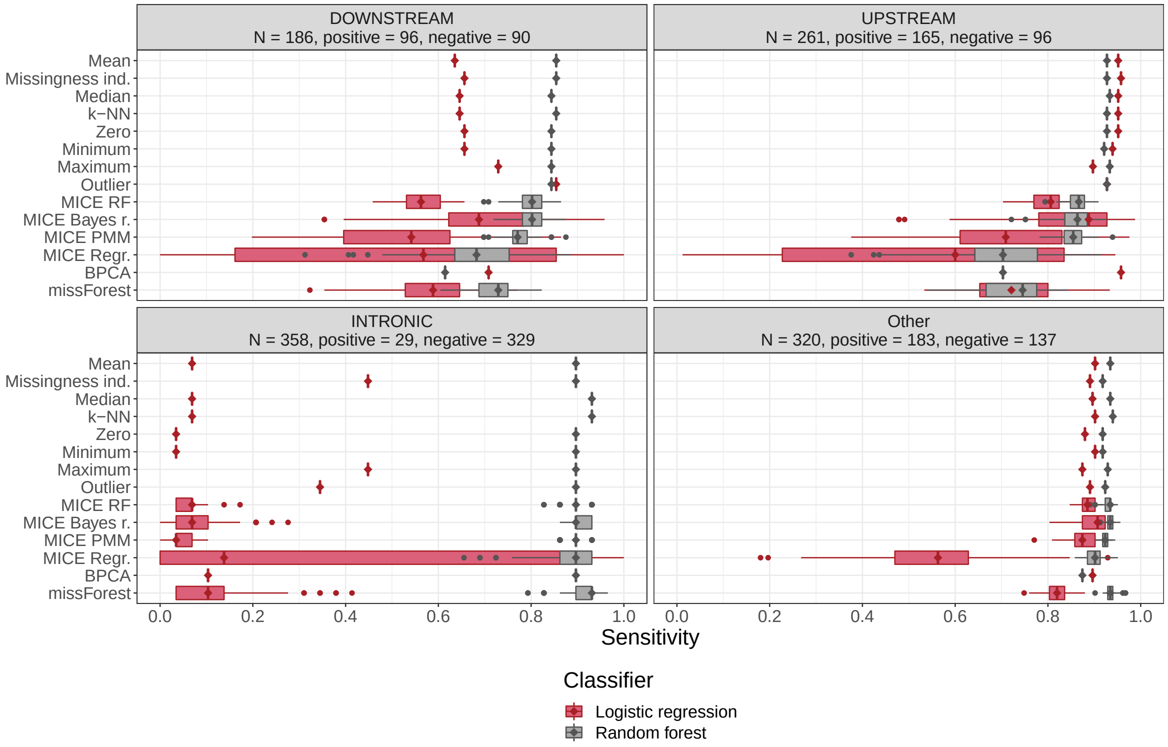

Supplementary Figure S7: Classifier sensitivity in the Main experiment, conditional on variant consequence. Classifier sensitivity on the test set is represented with boxplots (horizontal axis) per imputation method (vertical axis), stratified by predicted variant consequence. Diamonds were added to emphasize median values.

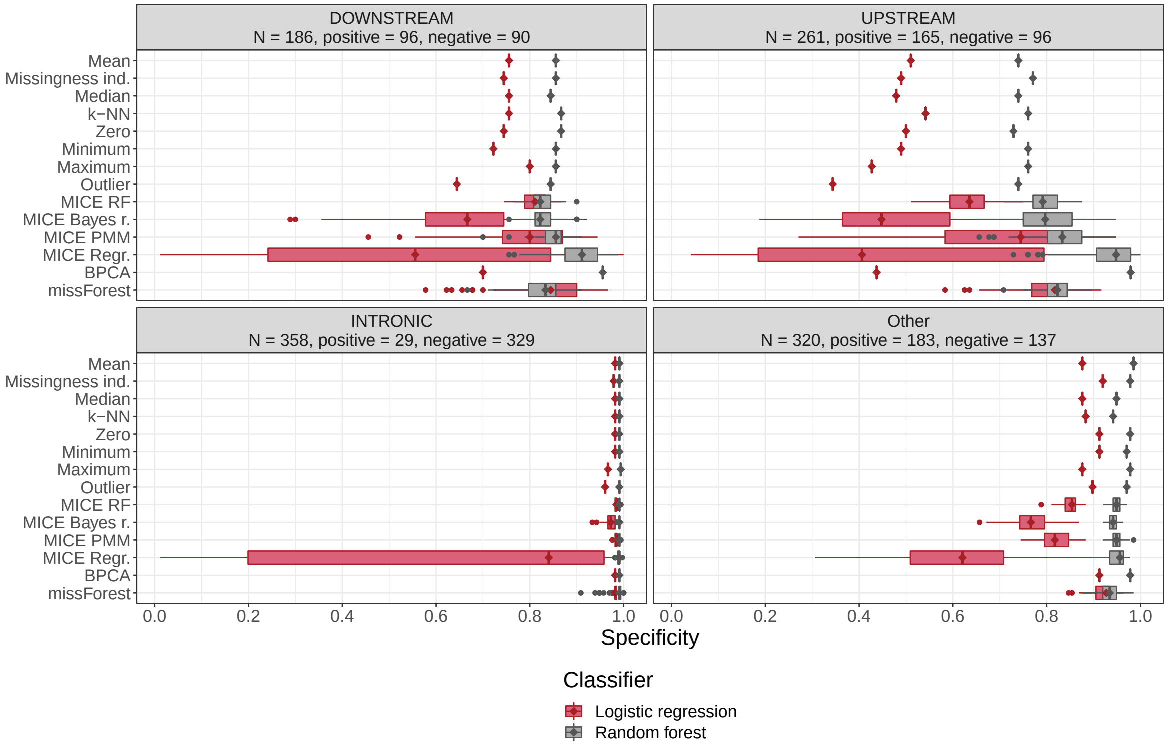

Supplementary Figure S8: Classifier specificity in the Main experiment, conditional on variant consequence. Classifier specificity on the test set is represented with boxplots (horizontal axis) per imputation method (vertical axis), stratified by predicted variant consequence. Diamonds were added to emphasize median values.

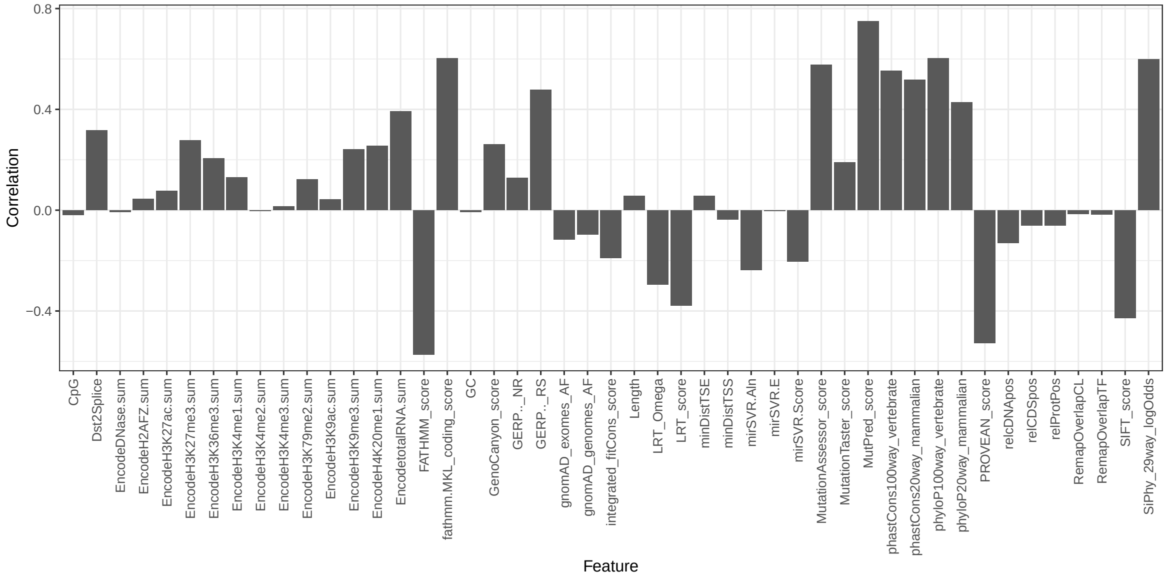

Supplementary Figure S9: Correlations of each feature to the positive outcome indicator. Correlation is shown (vertical axis) for each feature (horizontal axis).

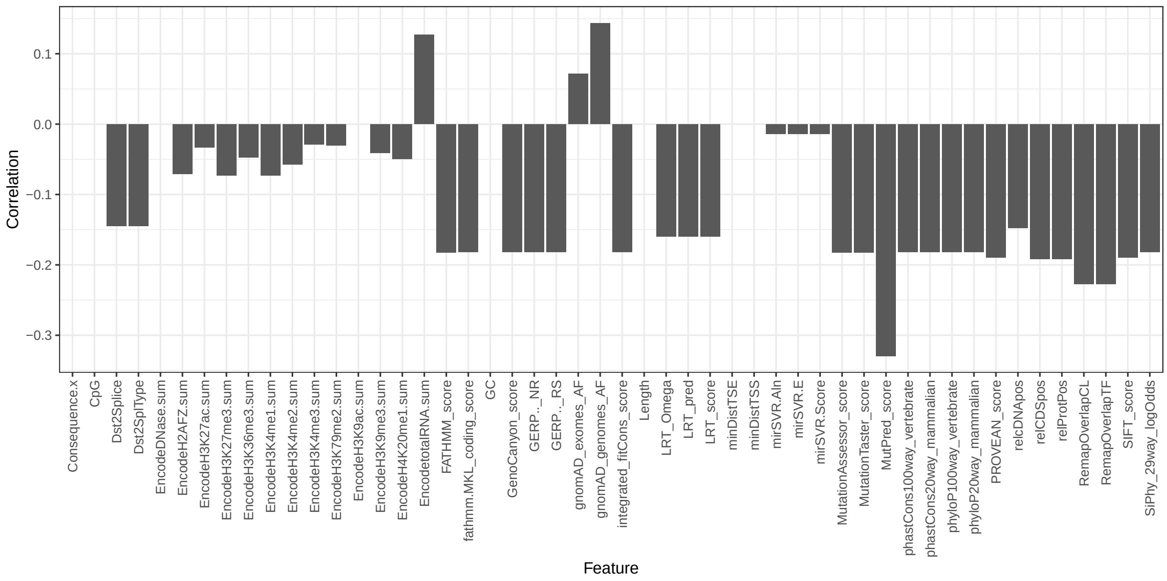

Supplementary Figure S10: Correlations of each feature's missingness indicator to the positive outcome indicator. Correlation is shown (vertical axis) for each feature (horizontal axis).

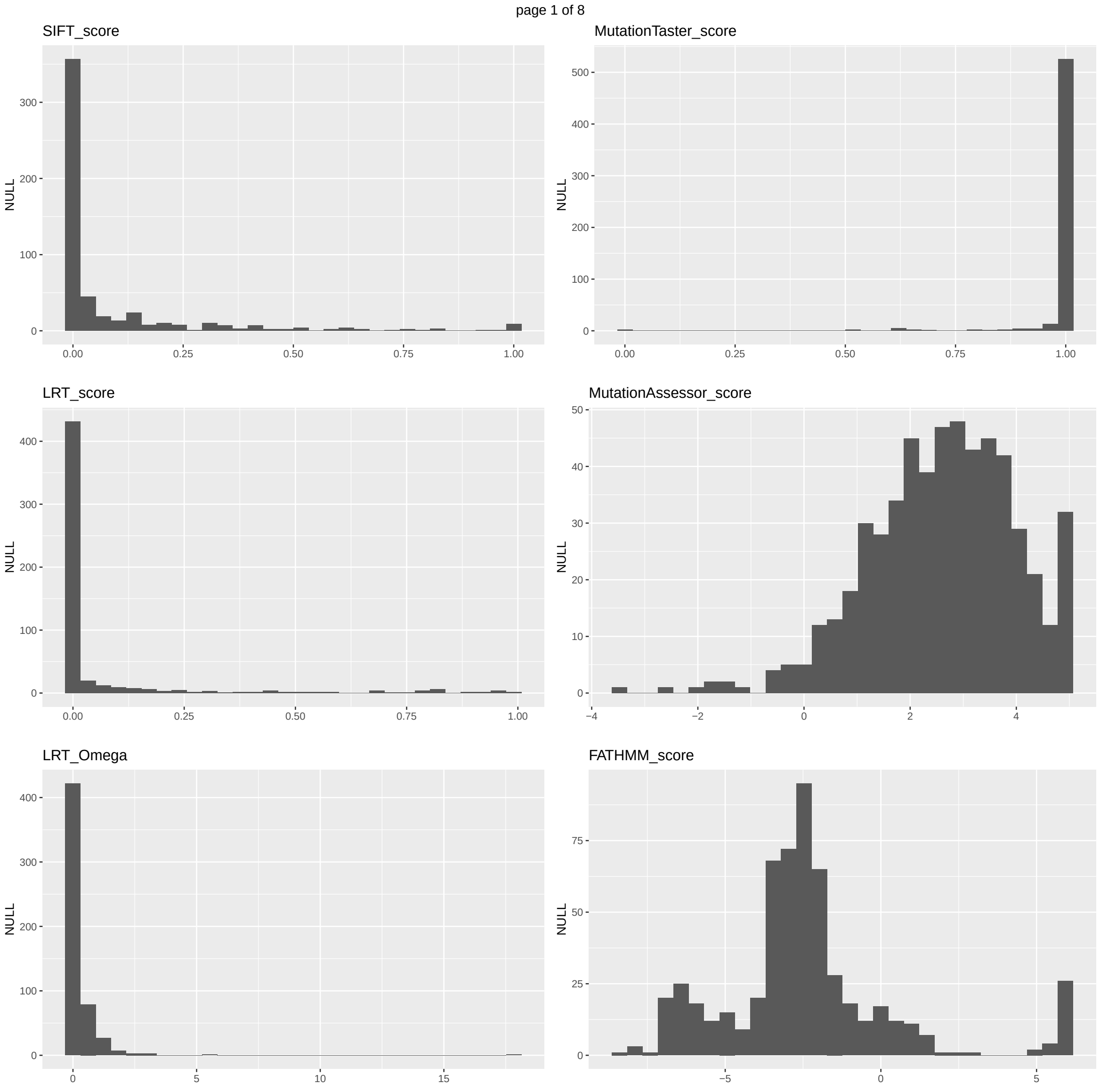

Supplementary Figure S11: Histograms of the observed values of features, page 1.

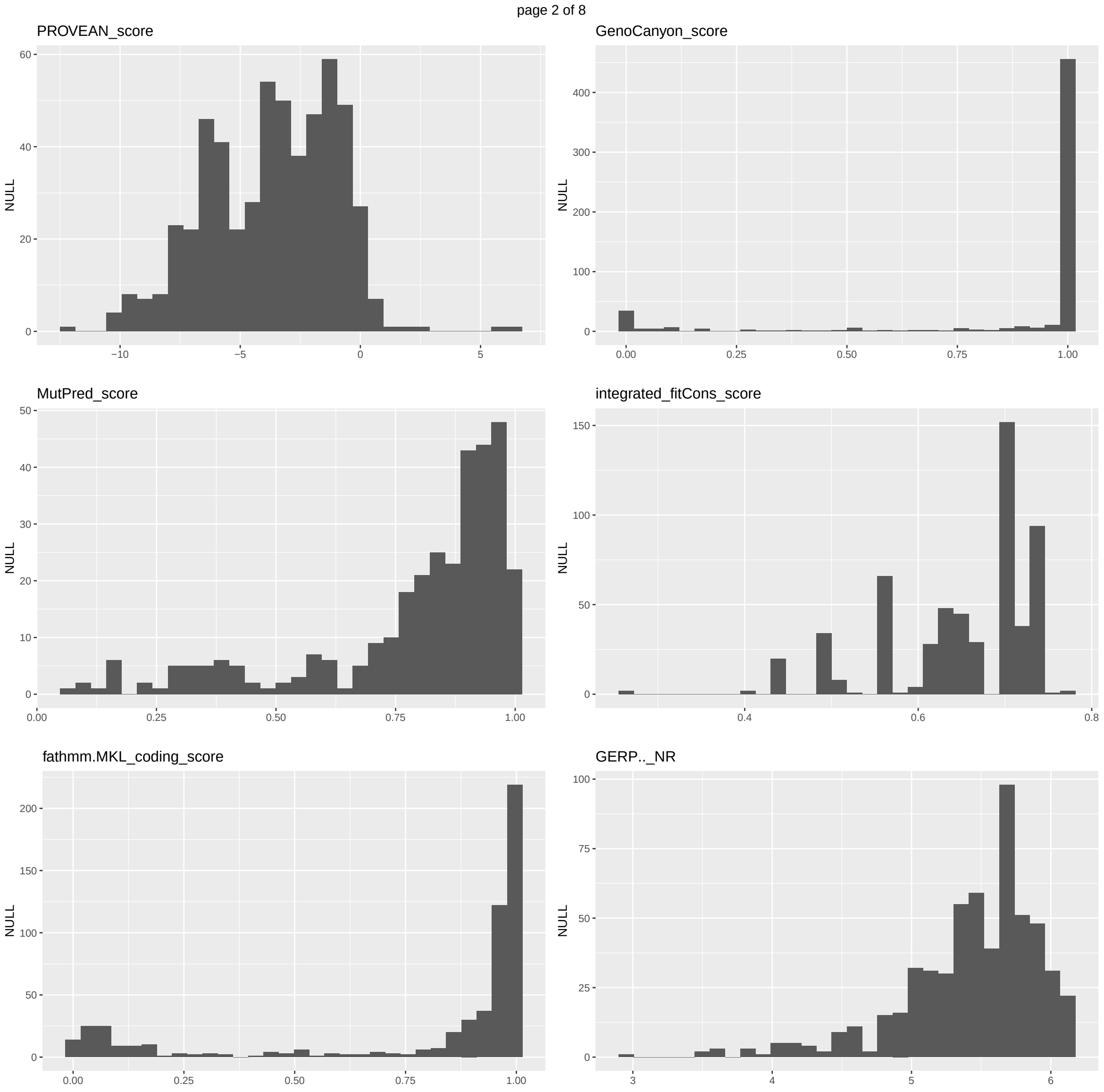

Supplementary Figure S12: Histograms of the observed values of features, page 2.

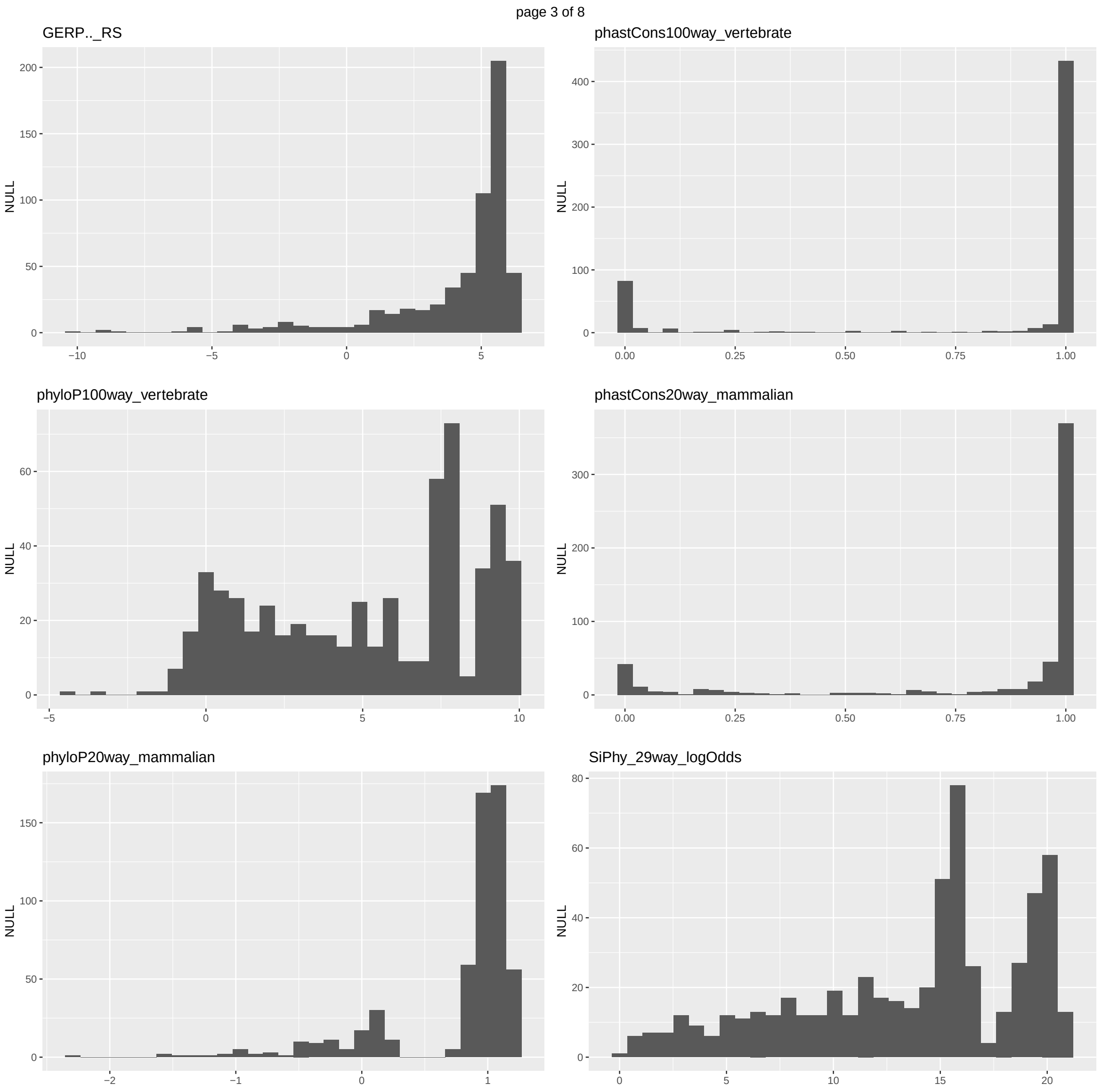

Supplementary Figure S13: Histograms of the observed values of features, page 3.

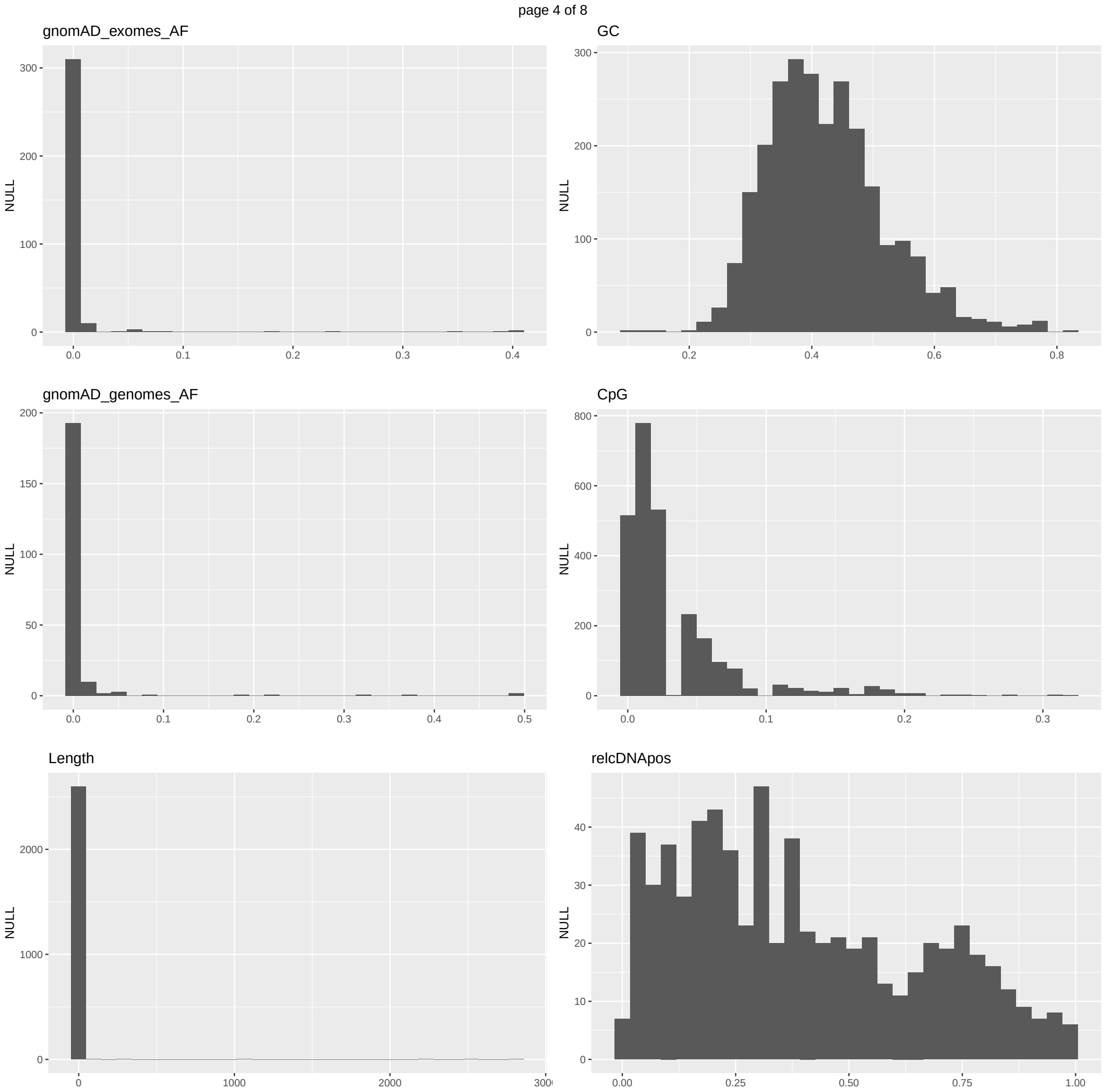

Supplementary Figure S14: Histograms of the observed values of features, page 4.

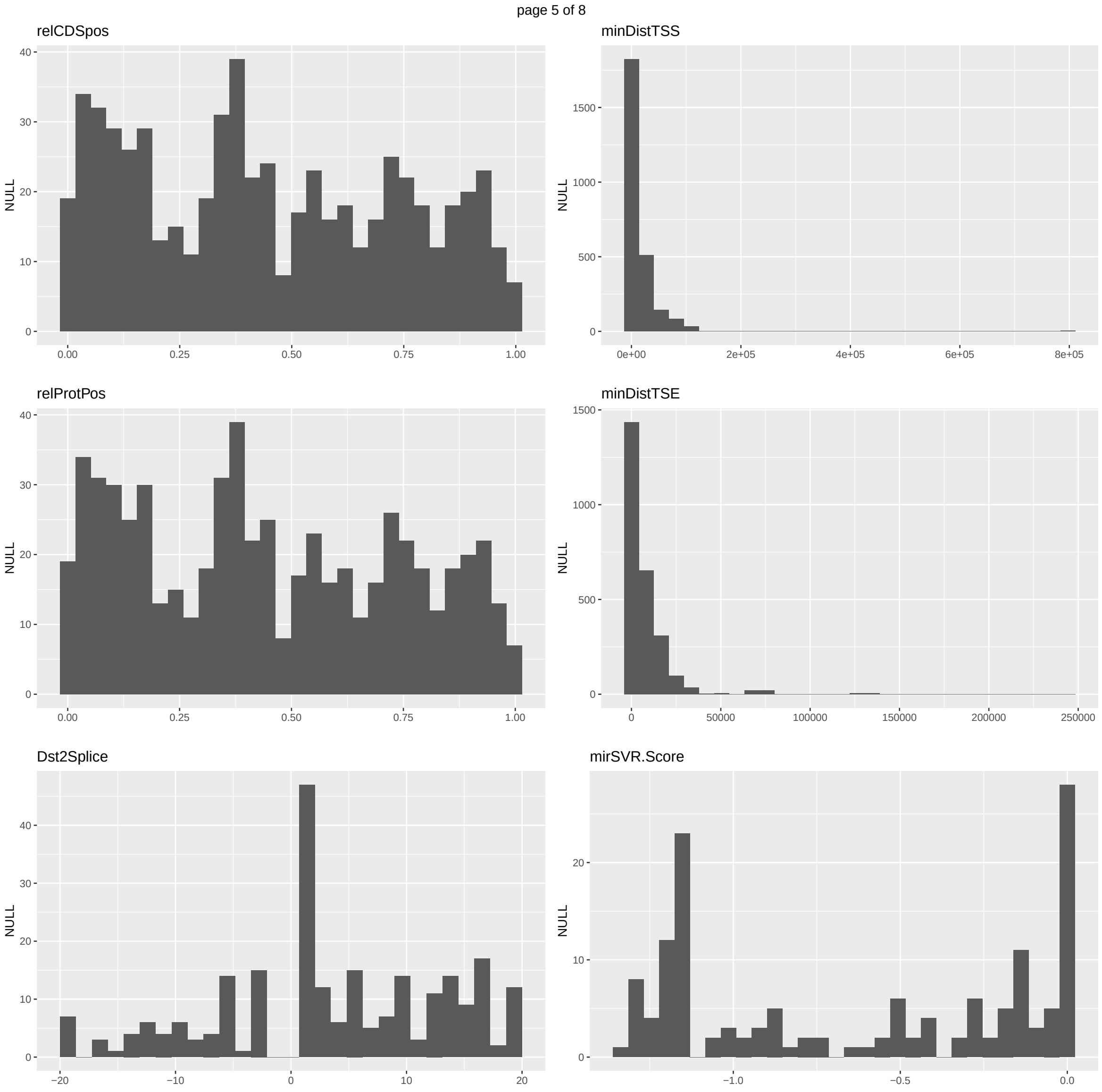

Supplementary Figure S15: Histograms of the observed values of features, page 5.

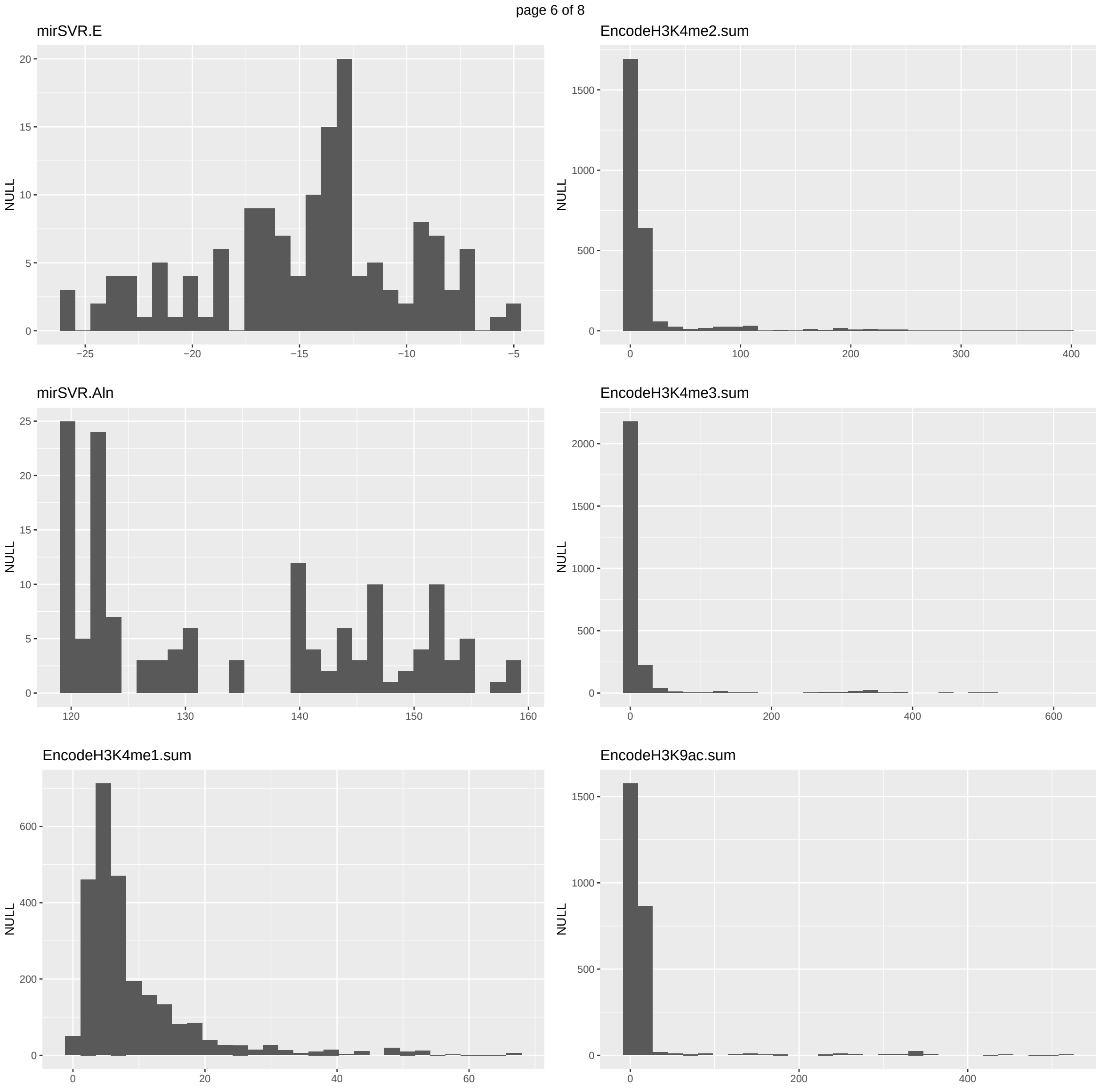

Supplementary Figure S16: Histograms of the observed values of features, page 6.

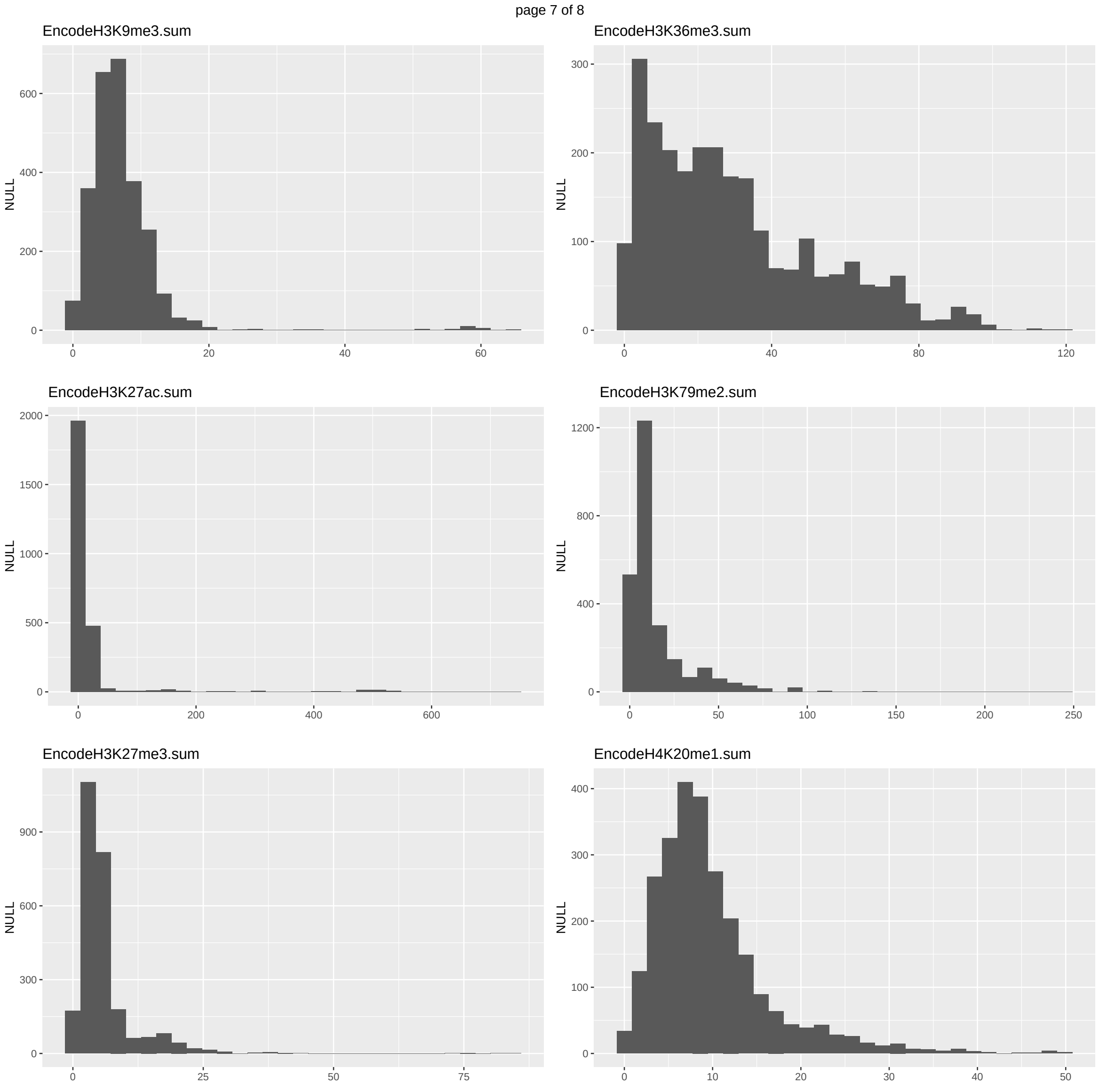

Supplementary Figure S17: Histograms of the observed values of features, page 7.

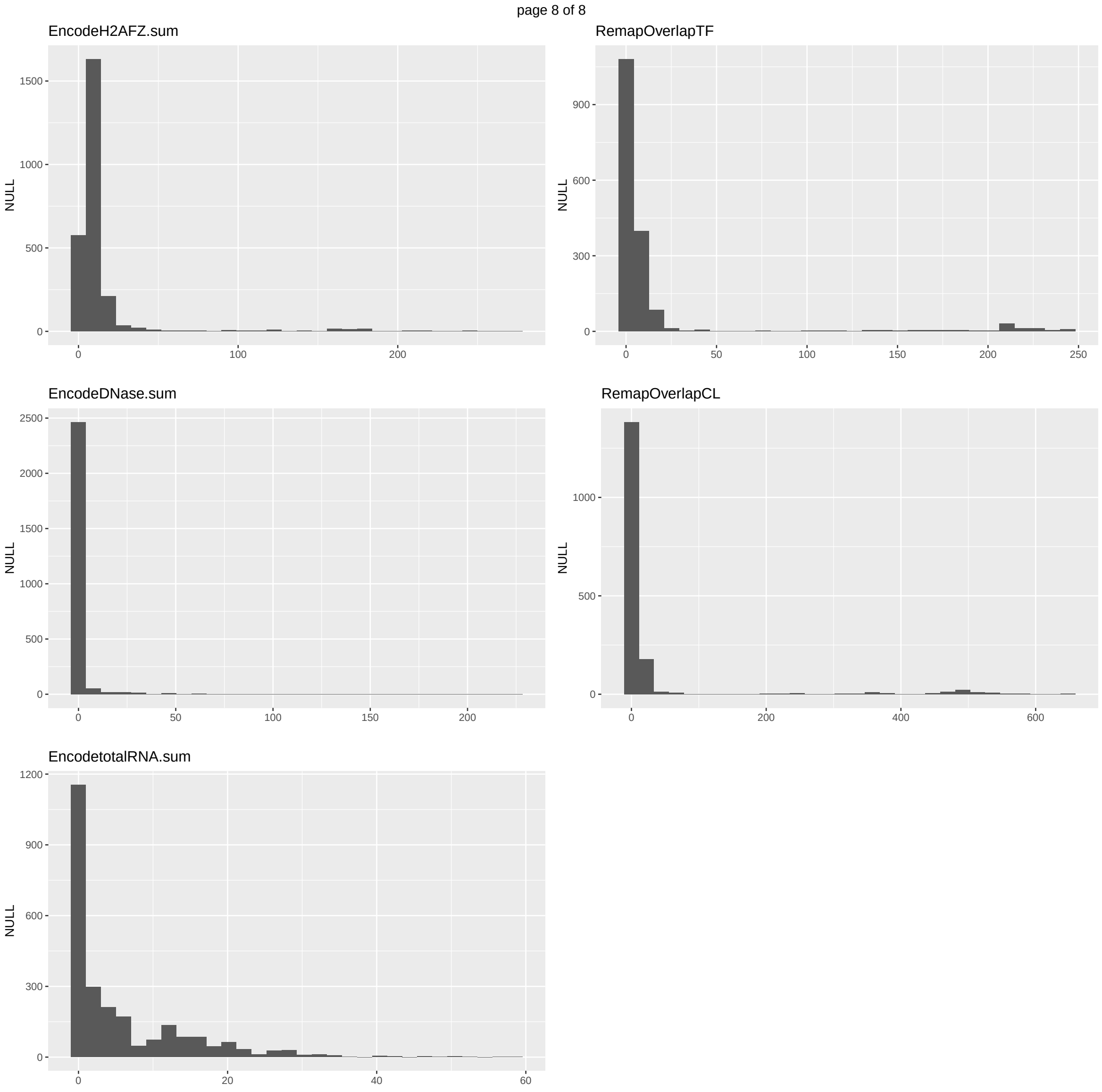

Supplementary Figure S18: Histograms of the observed values of features, page 8.

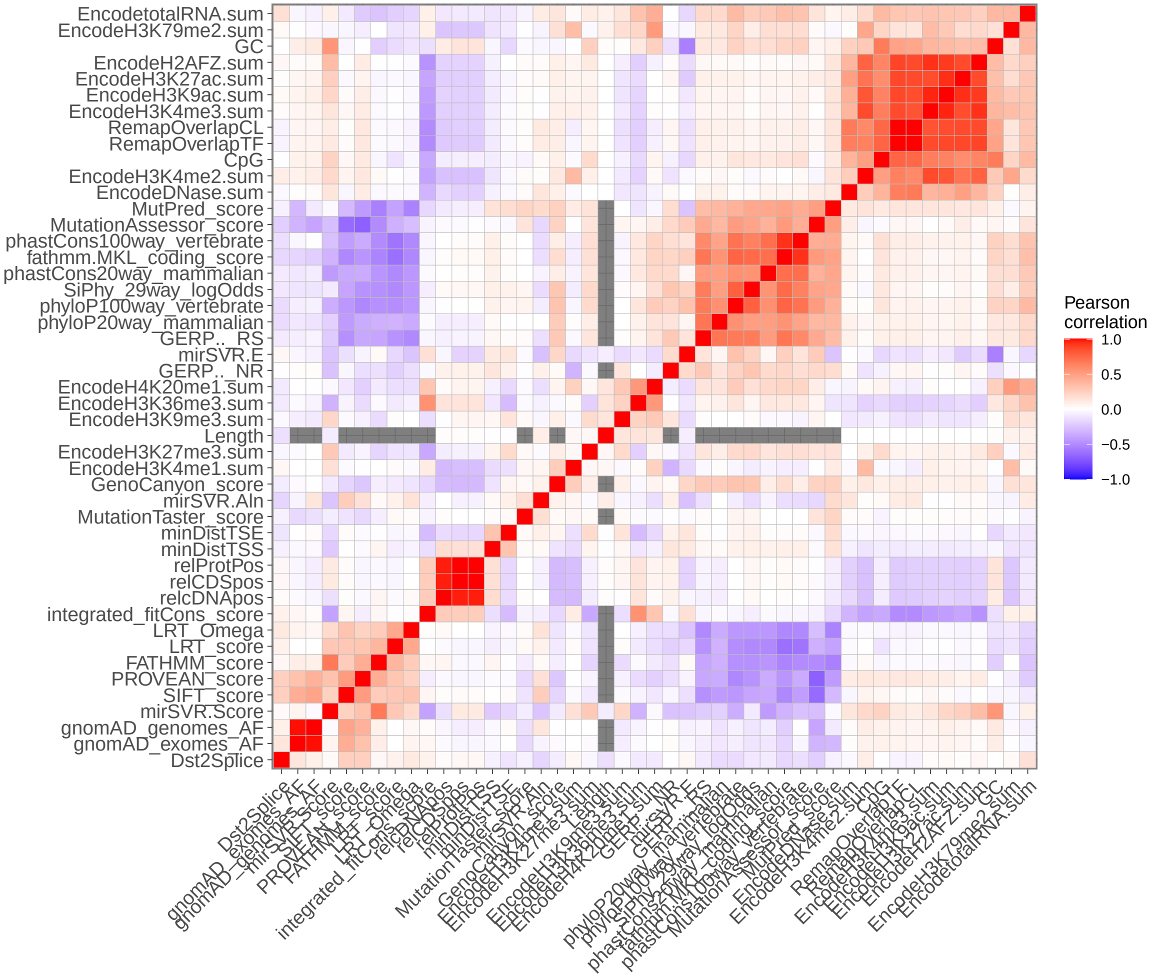

Supplementary Figure S19: Heatmap plot of the Pearson correlations between numeric features in the training data. Hierachical clustering was used to order the features. Empty cells (in grey) represent missing correlations arising from variables not being observed at the same time in any data point.
